# Social status regulates the hepatic miRNAome in rainbow trout: Implications for posttranscriptional regulation of metabolic pathways

**DOI:** 10.1371/journal.pone.0217978

**Published:** 2019-06-13

**Authors:** Daniel J. Kostyniuk, Dapeng Zhang, Christopher J. Martyniuk, Kathleen M. Gilmour, Jan A. Mennigen

**Affiliations:** 1 Department of Biology, University of Ottawa, Ottawa, Ontario, Canada; 2 Department of Biology, Saint Louis University, Saint Louis, Missouri, United States of America; 3 Department of Physiological Sciences and Center for Environmental and Human Toxicology, UF Genetics Institute, College of Veterinary Medicine, University of Florida, Gainesville, FL, United States of America; Universidade de Vigo, SPAIN

## Abstract

Juvenile rainbow trout develop social hierarchies when held in dyads, and the development of socially subordinate (SS) and social dominance (SD) phenotypes in this context has been linked to specific changes in the hepatic energy metabolism of all major macronutrients. Following our recently reported finding that transcript abundance of *drosha*, a key component of the microRNA (miRNA) biogenesis pathway, is increased in paired juvenile rainbow trout irrespective of social status compared to socially isolated (SI) controls, we here determined global changes of the hepatic miRNA pathway genes in detail at the transcript and protein level. Both socially SD and SS rainbow trout exhibited increased Ago2 protein abundance compared to SI rainbow trout, suggesting that hepatic miRNA function is increased in rainbow trout maintained in dyads. Given the well-described differences in hepatic intermediary metabolism between socially SD and SS rainbow trout, and the important role of miRNAs in the posttranscriptional regulation of metabolic pathways, we also identified changes in hepatic miRNA abundance between socially SS and SD rainbow trout using small RNA next generation sequencing. We identified a total of 24 differentially regulated miRNAs, with 15 miRNAs that exhibited increased expression, and 9 miRNAs that exhibited decreased expression in the liver of socially SS trout compared to socially SD trout. To identify potential miRNA-dependent posttranscriptional regulatory pathways important for social status-dependent regulation of hepatic metabolism in rainbow trout, we used an *in silico* miRNA target prediction and pathway enrichment approach. We identified enrichment for pathways related to metabolism of carbohydrates, lipids and proteins in addition to organelle-specific processes involved in energy metabolism, especially mitochondrial fusion and fission. Select predicted miRNA-mRNA target pairs within these categories were quantitatively analyzed by *real-time* RT-PCR to validate candidates for future studies that will probe the functional metabolic roles of specific hepatic miRNAs in the development of socially SD and SS metabolic phenotypes.

## 1. Introduction

Juvenile salmonid fish establish linear dominance hierarchies as a result of competition for shelter and feeding territories [1–3]. Socially dominant (SD) fish within these hierarchies monopolize preferred territories, displaying high levels of aggression towards their socially subordinate (SS) counterparts [1,3]. These differences in behaviour are accompanied by a range of physiological responses, including changes in energy metabolism [4–7]. Previous studies revealed an increased potential for hepatic glucose liberation in SS compared to SD fish. SS trout displayed increased mobilization of stored glycogen compared to SD trout, as evidenced by lower hepatic glycogen concentrations and higher glycogen phosphorylase activity [6]. Furthermore, SS trout displayed enhanced gluconeogenic and decreased glycolytic potential [4], supported by increased hepatic phosphoenolpyruvate carboxykinase (Pck) activity and decreased pyruvate kinase (Pk) activity [4]. These changes are in part dependent on the glucocorticoid stress hormone cortisol [4–10], with chronically elevated cortisol levels in SS trout leading to increased circulating glucose concentrations [10]. However, although circulating glucose represents an important fuel source for specific rainbow trout tissues, such as the brain [11], glucose utilization for global energy metabolism in most other tissues is limited in rainbow trout [12]. In contrast, lipid metabolism is a key player in global energy metabolism in trout [7], and SS trout exhibit increased reliance on free fatty acids, as indicated by elevated circulating free fatty acid concentrations at the organismal level, and by increased expression of the mitochondrial free fatty acid transporter carnitine palmitoyltransferase (*cpt1a*) which is rate limiting to mitochondrial β-oxidation [7]. Conversely, SD trout reveal increased capacity for hepatic *de novo* lipogenesis, as indicated by increased abundance of the transcription factor sterol regulatory element binding protein 1c (*srebp1c*) and the enzyme fatty acid synthase (*fasn)* mRNA, which coincide with increased circulating levels of triglycerides [7]. Finally, recent circumstantial evidence suggests that hepatic protein metabolism may be affected by social status, because increased activated ribosomal protein S6, which is associated with increased protein translation, was observed in the liver of SS fish [7].

Over the last decade, posttranscriptional regulation has emerged as an important mechanism for control of hepatic energy metabolism largely in mammalian models [13], but also in teleost fishes, especially rainbow trout, where hepatic miRNAs have been shown to regulate carbohydrate and lipid metabolism [14,15]. In a recent study, we determined that the transcript abundance of *drosha*, a key component in canonical miRNA biogenesis [16], is upregulated in liver of both SD and SS trout relative to socially isolated (SI) fish, which were handled similarly but housed without a conspecific [7]. Here we investigated the expression of several key canonical miRNA biogenesis pathway components, including *ago2* and *xpo5* paralogues, in more detail. Following additional evidence that key components of the canonical miRNA biogenesis pathway were increased in both SD and SS rainbow trout compared to SI rainbow trout strongly suggesting a social status dependent function of hepatic miRNAs, we measured differential hepatic miRNA expression between SS and SD rainbow trout using a next generation sequencing (NGS) approach. By means of an *in silico* pipeline, we identified several metabolic pathways that potentially are post-transcriptionally regulated by the differentially expressed miRNAs, and measured expression of specific miRNAs-mRNA pairs to prioritize targets for future functional analysis.

## 2. Materials and methods

### 2.1 Experimental animals

All experimental animals used in the current study have been previously described [7]. Care was taken to use the same initial weight between SI fish (100.14 g ± 5.10 g) and paired fish groups (100.92 g± 3.46 g). Briefly, juvenile rainbow trout, *Oncorhynchus mykiss*, were purchased from Linwood Acres Trout Farm (Campbellcroft, ON, Canada) and maintained in 1275 L fibreglass tanks at the University of Ottawa Aquatic Facility. All tanks were connected to flowing, aerated and dechloraminated 13°C city of Ottawa tap water. Trout were held under a 12L:12D photoperiod and acclimated to these holding conditions for a minimum of 2 weeks prior to experimentation. All fish were fed a ration equivalent to 0.5% body mass daily by distributing commercial trout pellets (Zeigler Finfish Silver, Gardners, PA, USA) in the tank. The holding of large groups (>20 trout), as well as animal care procedures (e.g. use of scatter feeding, homogenous tanks with a mild current) were employed to minimize hierarchy formation prior to experimentation. To establish social status, fish were lightly anaesthetized in a solution of benzocaine (0.05 g L^-1^ ethyl-*p*-aminobenzoate; Sigma-Aldrich, Oakville, ON, Canada) and mass and fork length and fin damage were quantified as previously described [4–7,10]. In cases where morphological differences did not allow for identification of individual fish, a pectoral fin clip was used for identification. Fish dyads were then established taking care not to exceed 5% of fork length differences between fish in a pair. After the initial assessment, pairs were placed in a 40 L flow-through Plexiglas observation tank, in which individuals were separated by an opaque, perforated divider. Tanks were maintained with flowing aerated and dechloraminated 13°C city of Ottawa tap water. The next morning, dividers were removed and fish were allowed to interact for a period of 4 d. At the end of the first day, a shelter in the form of a T-shaped PVC tube (11 x 13 cm long, 6 cm diameter) was added to the tanks. Behavioural observations were carried out twice per day between 9:00–11:00 h and 15:00–17:00 h, respectively. Each individual observation period within these time periods consisted of 5 min.

**Fig 1:**
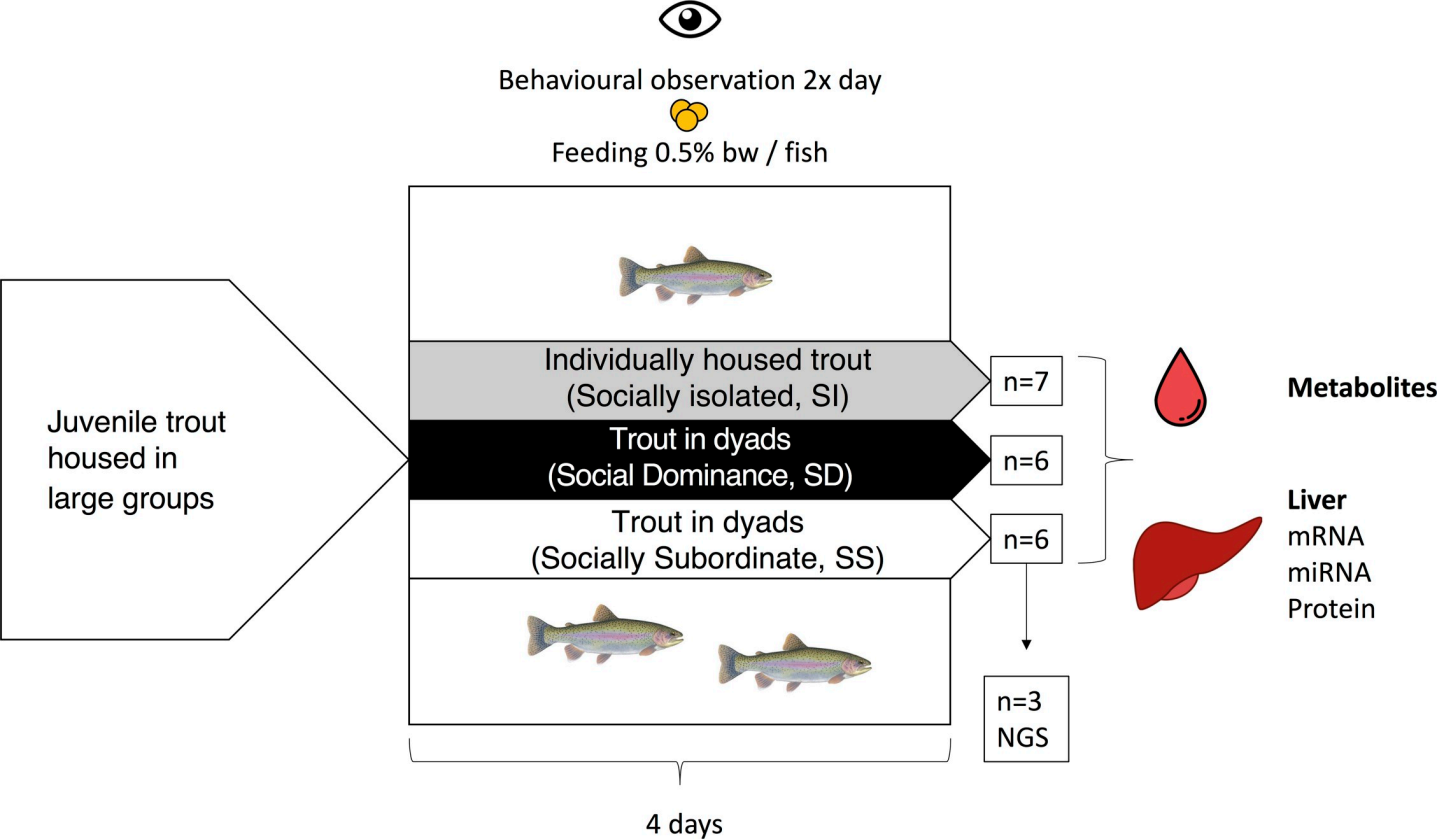
Schematic representation of the experimental design used to investigate the role of hepatic miRNAs in social status-dependent intermediary metabolism in juvenile rainbow trout, *Oncorhynchus mykiss*. *bw*, body weight; *NGS*, next generation sequencing.

The group of SI rainbow trout was subjected to the same handling and treatment procedures as paired fish, but were housed individually in the Plexiglas observation tanks. All fish were offered 0.5% fish mass per tank daily following the final observation period (except on the initial day of interaction), and the mass of food consumed as well as the fish that consumed the food were noted. A summary of the experimental design is shown in **Fig 1**. Social status was assessed as previously described (4–7,10) by assigning points to each fish for position within the tank, food acquisition, aggressive acts, and fin damage acquired during the 4 d interaction period. The applied scoring system awards more points for more SD behaviours, specifically patrolling the water column in the tank, acts of aggressive behaviour, being the first to feed and absence of fin damage. A principle components analysis (SigmaPlot v13.0; Systat software, San Jose, CA, USA) was used to calculate behaviour scores for each fish based on the mean scores across observation periods of each parameter. Within a pair, the fish with the higher score was assigned SD status, while the fish with the lower score was SS.

Specific behaviour scores as well as additional endocrine parameters associated with social status (plasma cortisol concentrations) allowed to clearly distinguish SD and SS fish in dyads, and detailed measurements of these parameters can be consulted in a previous publication [7]. For the purpose of the current study, it is important to note that two factors with particular importance to the social status-dependent metabolic phenotype exhibited clear differences. Firstly, while all fish nominally received 0.5% food rations, SD fish generally monopolized all food offered in a dyad, resulting in 1% food ratio for the SD individual, and *de facto* fasting for the SS individual over the 4 day period. Secondly, SS fish displayed a chronic activation of the endocrine stress axis, as evidenced by significantly increased cortisol concentrations compared to SD and SI fish [7].

Following the 4 d observation period, all fish in a pair were rapidly euthanized via terminal anesthesia (0.5 g L^-1^ ethyl-*p*-aminobenzoate; Sigma-Aldrich). Final mass and fork length were measured, fin damage scored, and blood samples collected via caudal venipuncture into heparin-coated syringes (2500 IU mL^-1^ heparin sodium salt; Sigma-Aldrich). All blood samples were centrifuged (10,000 *g* for 2 min) and plasma samples were flash frozen in liquid nitrogen before being stored at -80°C. Liver tissue was collected, freeze clamped, and stored at -80°C for subsequent analysis. All experimental protocols complied with the guidelines of the Canadian Council on Animal Care (CCAC) for the use of animals in research and teaching and were approved by the University of Ottawa’s Animal Care Committee under protocol number BL-2118.

### 2.2. Real-time RT-PCR assays for mRNA and miRNA

#### 2.2.1. Relative abundance mRNA quantification

Following the manufacturer’s protocol, total RNA was extracted from 20 to 100 mg of liver using TRIzol reagent (Invitrogen, Burlington, ON, Canada). To homogenize the tissues, the solution of TRIzol and tissue was forced through 18-G and 23-G needles using a syringe until the solution passed easily through the needle. Extracted RNA was quantified using a NanoDrop 2000c UV-Vis Spectrophotometer (Thermo-Fisher Scientific, Mississauga, ON, Canada) and RNA integrity was assessed using a Bioanalyzer (model 2100, Agilent Technologies, Mississauga, ON, Canada). Next, cDNA was generated using a QuantiTech Reverse Transcription Kit (Qiagen, Toronto, ON, Canada) following the manufacturer’s protocol which includes a DNA wipeout step before reverse transcription occurs. A noRT negative control sample containing water in place of the RT enzyme was also generated. Two-step relative abundance real-time RT-PCR assays were performed on a BioRad CFX96 instrument (Bio-Rad, Mississauga, ON, Canada) to quantify fold-changes in relative hepatic mRNA abundances of key transcripts involved in canonical miRNA biogenesis (*xpo5a*, *ago2a*, *ago2b*) and metabolic target genes (*pck1*, *pck2*, *g6pca*, *g6pcb1a*, *g6pcb1b*, *g6pcb2a*, *g6pcb2b*,*pygl*, *pygb*, *hsl*, *mfn1*, *mfn2*, *fis1*) as well as two reference genes (*ef1a* and *18s*). A standard curve consisting of serial dilutions of pooled cDNA, as well as a negative no-RT control consisting of cDNA generated in a reaction that did not include reverse transcriptase, and individual samples were run in duplicate for each experiment. The total volume was 20 μl, which consisted of 4 μl of diluted cDNA template, 0.5 μl of 10 nM specific forward and 0.5 μl of 10 nM specific reverse primer (**Table 1**), 10 μl of SsoAdvanced Universal Inhibitor-Tolerant SYBR Green Supermix (Bio-Rad), and 5 μl of H_2_O for each individual reaction. Real-time RT-PCR cycling parameters were a 5 min activation step at 95°C, followed by 40 cycles consisting of a 20 s denaturation step at 95°C and a 30 s annealing and extension step at a primer-specific temperature (**Table 1**). After each run, melting curves were produced and monitored for single peaks to confirm the specificity of the reaction and the absence of primer dimers. In cases where primers were newly designed (**Table 1**), purified and pooled PCR products were sent for sequencing (Ottawa Hospital Research Institute, Ottawa, ON, Canada), and a BLAST search (National Center for Biotechnology Information, Bethesda, MD, USA) was used to confirm amplicon specificity. All amplification efficiencies calculated from serially diluted 7-point standard curves were between 90–110%, with R^2^ values > 0.98. Relative transcript abundance derived from standard curves was normalized using the NORMA-Gene approach as described by Heckman et al. [17]. Afterwards, mRNA fold changes were calculated relative to the SI group and data were analyzed and plotted using Prism Version 7 (GraphPad Software, San Diego, CA, USA) and transformed to fit a normal distribution. Grubb’s outlier test was used to identify and remove single outliers in a treatment group. Data were subsequently analyzed using one-way analysis of variance (ANOVA) followed by Tukey’s post-hoc test.

**Table 1:** Real-time RT-PCR primer sequences and reaction parameters.

Gene target	Primer pair (5’ to 3’)	Annealing temperature (°C)	Efficiency (%)	R^2^	Reference
***Canonical microRNA biogenesis pathway***					
*xpo5a* GSONMT00065065001	F: AGTCAACTGGGTGGGGATTC R: TCCCACCTCAGACATGCTCA	59	104.1	0.98	
*xpo5b* GSONMT00007401001	F ACTCTCGAACACGGCTGATG R: CACACATATCAGCCACCGGT	59	103.6	0.98	
*ago2a* GSONMT00060791001	F: GTGGTGGGGTGGACTCATTT R: AAACCTTCAATCGGGCCCTT	59	107.5	0.99	
*ago2b* *GSONMT00025973001*	F : TGTCGCACGGTGTTAACATG R: AGCAATGCCGAGAAGAGCTA	58	99.3	0.98	
***microRNAs***					
*fru-miR-21a-5p*	F: TAGCTTATCAGACTGGTGTTGGC R: Taqman Universal Reverse Primer	60	95.7	0.98	
*fru-miR-722*	F: TTTTTTGCAGAAACGTTTCAGATT R: Taqman Universal Reverse Primer	60	98.1	0.99	
*ssa-miR-26a-5p*	F : TTCAAGTAATCCAGGATAGGCT R : Taqman Universal Reverse Primer	60	103.2	0.98	
*fru-miR-let-7a*	F: TGAGGTAGTAGGTTGTATAGTT R: Taqman Universal Reverse Primer	60	99.3	0.98	
*fru-miR-152*	F: TCAGTGCATAACAGAACTTTGT R: Taqman Universal Reverse Primer	60	107.9	0.99	
***Hepatic glucose metabolism***					
*pck1* *GSONMG00082468001*	F: ACAGGGTGAGGCAGATGTAGG R: CTAGTCTGTGGAGGTCTAAGGGC	55	104.1	0.99	
*pck2* *GSONMG00059643001*	F: ACAATGAGATGATGTGACTGCA R: TGCTCCATCACCTACAACCT	56	105.1	0.99	
*g6pca* *GSONMG00076843001*	F: GATGGCTTGACGTTCTCCT R: AGATCCAGGAGAGTCCTCC	55	91.0	0.98	[33]
*g6pcb1a* *GSONMG00076841001*	F: GCAAGGTCCAAAGATCAGGC R: GCCAATGTGAGATGTGATGGG	59	108.1	0.99	[33]
*g6pcb1b* *GSONMG00066036001*	F: GCTACAGTGCTCTCCTTCTG R: TCACCCCATAGCCCTGAAA	55	95.0	0.98	[33]
*g6pcb2a* *GSONMG00013076001*	F: ATCGGACAATACACACAGAACT R: CAACTGATCTATAGCTGCTGCCT	55	93.4	0.99	[33]
*g6pcb2b* *GSONMG00014864001*	F: CCTCTGCTCTTCTGACGTAG R: TGTCCATGGCTGCTCTCTAG	56	103.7	0.98	[33]
*pygl* *GSONMT00046884001*	F : TCAAGAACATCGCTGCGTCT R : TAACTTCACCCTGGTCACGC	59	100.3	0.99	
*pygb* *GSONMT00041162001*	F : GTGATCCCTGCAGCTGACTT R : TCCTCTACCCTCATGCCGAA	55	100.4	0.98	
***Hepatic lipid metabolism***					
*hsl* *GSONMT00023441001*	F : TGCCTTCCTGTACTCGCAAG R : GCAAGAAAGGCAAGGGTGTT	55	101.1	0.99	
***Mitochondrial dynamics***					
*mfn1* *GSONMT00027395001*	F: AGGGAGCTGGAGGGAGAAAT R: CCAAGACAGGCTCCAGTGAA	55	106.4	0.99	
*mfn2* *GSONMT00004526001*	F :ACTGCTCCGGAATAAGGCTG R: GTGGTCTTTTCGCGGTCAAC	55	106.5	0.99	
*fis1* *GSONMT00039888001*	F : GGTTTGGTTGGCATGGCAAT R: TTCTCATCTTGGGGAACGGC	55	95.1	0.99	
***Reference genes***					
*ef1a*	F: CATTGACAAGAGAACCATTGA R: CCTTCAGCTTGTCCAGCAC	56	91.9	0.99	[7]
*snoRNA-U23*	F :GCCCATGTCTGCTGTGAAACAAT R : Taqman Universal Primer	61	105.6	0.98	[7]

#### 2.2.2. Relative abundance miRNA quantification

miRNA cDNA was synthesized using TaqMan MicroRNA Reverse Transcription Kit (Life Technologies Inc., Burlington, ON, Canada) with 10 ng of total RNA per 15 μl RT reaction containing 0.15 μl 100mM dNTPs (with dTTP), 1.00 μl MultiScribe Reverse Transcriptase 50 U/μl, 1.50ul 10X Reverse Transcription Buffer, 0.19 μl RNAse Inhibitor 20U/μl, 4.16ul nuclease free water, 5ul total RNA and 3ul miRNA specific 5X RT primer following the manufacturer’s protocol. Two step real-time RT-PCR was used to validate the abundance of *fru-miRNA-21-5p*, *dre-miRNA-722*, *dre-miRNA-26a-5p*, *dre-let-7a*, *fru-miRNA-152* using the CFX96-Real-Time System- C1000 Thermal Cycler machine (BioRad, Mississauga, ON, Canada). Real-time RT-PCR reactions were prepared in a total volume of 20 μL per reaction, containing 1.00 μL Taqman Small RNA Assay 20X, 1.33 μL Product from RT reaction, 10.00 μL Taqman Universal PCR Master Mix (2X) with UNG and 7.67 μL nuclease free water. A no reverse transcriptase (noRT), where the RT was replaced with nuclease free water, and a no template (noTemp), where product from RT reaction was replaced with nuclease free water, were included during cDNA synthesis as controls for DNA contamination. Following an activation step at 50°C (2 min) to activate UNG and another activation step at 95°C (10 min) to activate the polymerase in the qPCR mix, two steps were repeated for 40 cycles including the denaturation at 95°C (15 s) and a combined annealing and extending step at 60°C (60 s). Assays were subsequently normalized using the NORMA-Gene approach as described by Heckman et al. [17] miRNA fold changes were calculated relative to the fasted group. Data were analyzed using Prism Version 7 (GraphPad Software, San Diego, CA, USA), and transformed to fit normal distribution when necessary. In normally distributed data, Grubb’s outlier test was used to identify and remove possible single outliers in a treatment group. Data were subsequently analyzed using a one-tailed Welch’s t-test. Significance was determined at a p<0.05 level.

### 2.3. Protein extraction and Ago2 protein quantification

Fractions (~100mg) of frozen livers (n = 6 per treatment group) were weighted into 1 mL of lysis buffer 150mM NaCl, 10 mM Tris HCl, 1 mM EGTA, 1mM EDTA (pH 7.4), 100 mM sodium fluoride, 4mM sodium pyrophosphate, 2mM sodium orthovanadate, 1% Triton X-100, 0.5% NP-40-IGEPAL (all Sigma-Aldrich), and a protease inhibitor cocktail (Roche, Basel, Switzerland) and homogenized using a sonicator model 100 (Thermo-Fisher Scientific, Mississauga, ON, Canada). Homogenates were centrifuged at 15000 g for 30 min at 4°C, and the recovered supernatant stored at -80°C. Protein concentrations were determined using the Bio-Rad protein assay kit (Bio-Rad) with BSA (bovine serum albumin) as the standard. Lysates (20 μg of the total protein) were subjected to SDS-PAGE and wet-transfer Blotting. Membranes were incubated with a rabbit polyclonal antibody raised against a human AGO2 epitope (Proteintech, Chicago, IL, USA) at 4°C overnight. The next day, following blocking and washing steps, membranes were incubated with an IRDye infrared secondary anti-rabbit antibody (LI-COR Biosciences, Lincoln, NE, USA). The bands were visualized by infrared fluorescence using the Odyssey Imaging System (LI-COR Biosciences) and quantified by Odyssey infrared Imaging System software (version 3.0, LI-COR biosciences) using total protein stain as normalization method. A single band of expected size was detected and data were analyzed using Prism Version 7 following transformation to fit a normal distribution. Grubb’s outlier test was used to identify and remove possible single outliers in a treatment group. Data were subsequently analyzed using a one-way ANOVA followed by Tukey’s post-hoc test. Significance was determined at a p<0.05 level.

### 2.4. Small RNA next generation sequencing and differential expression analysis

The quality of Trizol-extracted total RNA was confirmed by RNA Integrity Numbers between 8.3 and 9.6, using an Agilent Technologies 2100 Bioanalyzer. Small RNA sequencing was performed by LC Sciences (Houston, TX, USA) using three randomly selected samples from each treatment group. Nucleotide fractions (15–50 nt) of small RNAs were isolated from the total RNA using polyacrylamide gel electrophoresis and were ligated to a 30 nt adapter followed by a 50 nt adapter (Illumina, San Diego, CA, USA). The small RNA ligated to the adaptors was reverse transcribed to cDNA, PCR amplified, and gel purified. The gel-extracted cDNA was used for library preparation, followed by cluster generation on Illumina's Cluster Station before sequencing using Illumina GAIIx. Raw sequence data were obtained from image data using Illumina's Sequencing Control Studio software version 2.8 following real-time sequencing image analysis and base-calling by Illumina's Real-Time Analysis version 1.8.70, and were deposited in the NCBI Gene expression Omnibus (GEO) under accession number GSE112815 with specific files (n = 3) for SS (GSM3084233-GSM3084235) and SD (GSM3084236-GSM3084238) rainbow trout liver samples.

Briefly, sequences were initially filtered with the proprietary LC Science ACGT10-miR v4.2 pipeline to remove low quality sequences, low complexity sequences, and sequences corresponding to common RNA families (mRNA, RFam, Repbase, piRNA), as described by Ma and colleagues [18]. Retained high quality sequences were then submitted to a bowtie search against all fish miRNA gene sequences deposited in miRbase v21.0 (1 mismatch allowed in the first 16 nt) and compared to published miRNAs from rainbow trout [19–21]. In cases where unique sequences mapped to the miRbase-deposited fish miRNA genes, the obtained miRbase hits were subsequently mapped to the rainbow trout genome to determine genomic locations (**S1 Table**). Any alignment that had already previously been characterized as a genomic location for this specific microRNA in the rainbow trout genome (**Table 2**) was identified as known rainbow trout miRNA (gp1a). All miRNAs that were successfully aligned to miRbase deposited fish species miRNA genes, and had not been described in the rainbow trout miRNA repertoire, but mapped to the rainbow trout genome, were retained as trout microRNAs (gp1b). The remaining miRbase hits from both gp1a and gp1b that in addition to known miRNA loci also mapped to other rainbow trout genome loci were designated as gp1c.

**Table 2:** Reference databases used for rainbow trout miRNA annotation.

Reference databases	WEBlink and Information	Version or Built Date
miRNA (miRs) database	ftp://mirbase.org/pub/mirbase/CURRENT/; Specific species: dre; Selected species: fru, ola, tni, ccr, hhi, ipu, pol, ssa	v21
Pre-miRNA (mirs) database	ftp://miRbase.org/pub/mirbase/CURRENT/; Specific species: dre; Selected species fru, ola, tni, ccr, hhi, ipu, pol, ssa	v21
Genome database	http://www.genoscope.cns.fr/trout/data/Oncorhynchus_mykiss_chr.fa.gz	04/28/2014
mRNA database	http://www.genoscope.cns.fr/trout/data/Oncorhynchus_mykiss_mRNA.fa.gz	04/28/2014

High quality sequences that could be matched to fish species miRNA genes in miRbase, which in turn could not be mapped to the rainbow trout genome, were further analyzed as follows: The high quality short sequence was directly mapped onto the rainbow trout genome, and the locus further analyzed for its possibility to generate hairpin transcripts. This was achieved by retrieving 80 nt flanking sequences of mapped high quality sequences. Retrieved sequence were then analyzed for their propensity to form hairpin structures using RNA-fold software (http://rna.tbi.univie.ac.at/cgi-bin/RNAfold.cgi) with the following criteria: (1) ≤ 12 nt in one bulge in stem; (2) ≥ 16 bp in the stem region of the predicted hairpin; (3) ≤ -15 kCal/mol cutoff of ΔG; (4) ≥ 50 hairpin length which contains up- and down stems and hairpin loop; (5) a ≤ 200 nt hairpin loop length; (6) ≤4 nt in one bulge in the mature miRNA region; (7) ≤2 errors in one bulge in mature region; (8) ≤2 biased bulges in mature region; (9) ≤4 errors in the mature miRNA region;(10) ≥ 12 bp in the mature region of the predicted hairpin; (11) >80% of mature miRNA is in located in the stem. Sequences in located in loci predicted to form hairpins were grouped as gp2a, and sequences mapped to loci not predicted to form hairpins were designated as gp2b. High quality sequences mapped to fish miRNA genes deposited in miRbase, which did neither map to the rainbow trout genome as fish miRbase gene hit, nor as mature sequence, but had been identified as mature miRNA in rainbow trout were designated gp3a. Conversely, high quality sequences mapped to fish miRNA genes deposited in miRbase, which did neither map to the rainbow trout genome as fish miRbase gene hit, nor as mature sequence, and had not been identified as mature miRNA in rainbow trout were designated gp3b. Finally, remaining sequences that could not be matched to miRbase fish species, but could be mapped to the rainbow trout genome, were again tested for the potential to form hairpin structures with flanking sequences. Sequences predicted to be able to form hairpins were designated as gp4a, sequences predicted to be unable to form hairpins were designated as gp4b. Details of the miRNAs and their respective groups are presented in **S2 Table**. Differentially expressed miRNAs were identified using the statistical software R (Version 3.2.2) package DESeq2 for t-test comparisons of SD versus SS groups.

### 2.5. In silico miRNA-target analysis

To predict rainbow trout specific mRNA targets of differentially regulated miRNAs, we utilized the miRanda package initially developed in the Enright lab [22]. The miRanda algorithm places emphasis on seed match and free energy of the duplex structure, two of the most relevant aspects in miRNA-target interaction [23–24]. Among the available miRNA target prediction algorithms [25–26], we chose the miRanda algorithm based on its increased sensitivity compared to other predictive algorithms [27], as well as its previous application across several rainbow trout transcriptome level miRNA target prediction studies [28,29], in an effort to facilitate comparison among studies. Because increased sensitivity occurs at the cost of an increased rate of false positive predictions, we used a stringent cut-off with a miRanda pairing score S > 140, and a free-energy score ΔG cut-off < -20, where S is the sum of single residue-pair match scores over the alignment trace, and ΔG is the free energy of duplex formation calculated by the Vienna package [30]. Annotated 3’UTRs were taken from the microTrout database [21].

### 2.6. Gene ontology enrichment, pathway, and sub-network enrichment analysis

Gene ontology enrichment of transcripts predicted to be targeted by differentially regulated miRNAs is a widely used approach to infer biological responses at the genome regulation level in rainbow trout and mammalian models [28,29,31]. We performed gene ontology enrichment in JMP Genomics V8.1. Parametric analysis of gene expression (PAGE) was conducted to determine which gene ontologies were significantly enriched in the dataset based upon predicted targets of miRNAs. The rainbow trout genome was used as a background list for comparison. Genes were considered to be miRNA targets if transcript-miRNA was predicted by the miRanda algorithm with cut-off values of S > 140 and ΔG < -20. The p-value for each gene ontology was corrected using the false discovery rate. To build pathways for gene targets, we imported the short list of genes within a group of gene ontology categories (i.e. those related to “glucose metabolism”) into Pathway Studio 11.4 (Elsevier, Amsterdam, Netherlands). The software uses known relationships (i.e. based on expression, binding, common pathways) between genes to create networks focused around a cell process. The miRNA targets were imported into the program using “Name + Alias” and rainbow trout genes were mapped to their mammalian homologues. Lastly, the miRNA targets were subjected to a subnetwork enrichment analysis in Pathway Studio for “Cell Process”. This approach was used to achieve a general overview of what cell processes were those most relevant to hepatic regulation of glucose and intermediary metabolism based on the miRNA targets. These biological functions are those most likely controlled post-transcriptionally by the regulated miRNA network.

### 2.7. Correlative analysis of miRNA-mRNA targets

Specific mRNA targets belonging to enriched pathways that were predicted to be regulated by differentially expressed miRNAs were quantified to elucidate a potential involvement in the physiological consequences of social interactions in the liver of rainbow trout, and to determine correlations between expression of miRNA and targeted mRNA transcripts. Although this approach does not indicate a functional relationship, such correlations act to prioritize specific miRNA-mRNA target pairs for future functional studies. The strength of correlations between differentially expressed miRNAs and the expression of their predicted specific targets was assessed using the Pearson correlation coefficient computed in Prism Version 7 (GraphPad Software, San Diego, CA, USA).

### 2.8. Glucose assay

Plasma glucose concentration in SD and SS fish were measured indirectly using a Spectra Max Plus384 Absorbance Microplate reader (Molecular Devices, Sunnyvale, CA, USA). Briefly, G6P-DH, NAD+ and ATP (all Sigma-Aldrich) were added to each undiluted plasma sample and a background measurement was taken. Hexokinase (Sigma-Aldrich) is then added, and the solution was incubated at room temperature for 30 min before measurement. Collectively, D-Glucose is converted to G-6-P by hexokinase and then to 6-phosphogluconate by G6P-DH and the resulting NADH allows for the indirect colorimetric measurement of glucose concentration. All spectrophotometric measurements were taken at 340nm. Data were normally distributed, but not homoscedastic, and were analyzed by Welch’s t-test using Prism Version 7 (GraphPad Software, San Diego, CA, USA).

## 3. Results

### 3.1. Induction of hepatic canonical miRNA biogenesis pathway components in a trout social hierarchy

**Fig 2:**
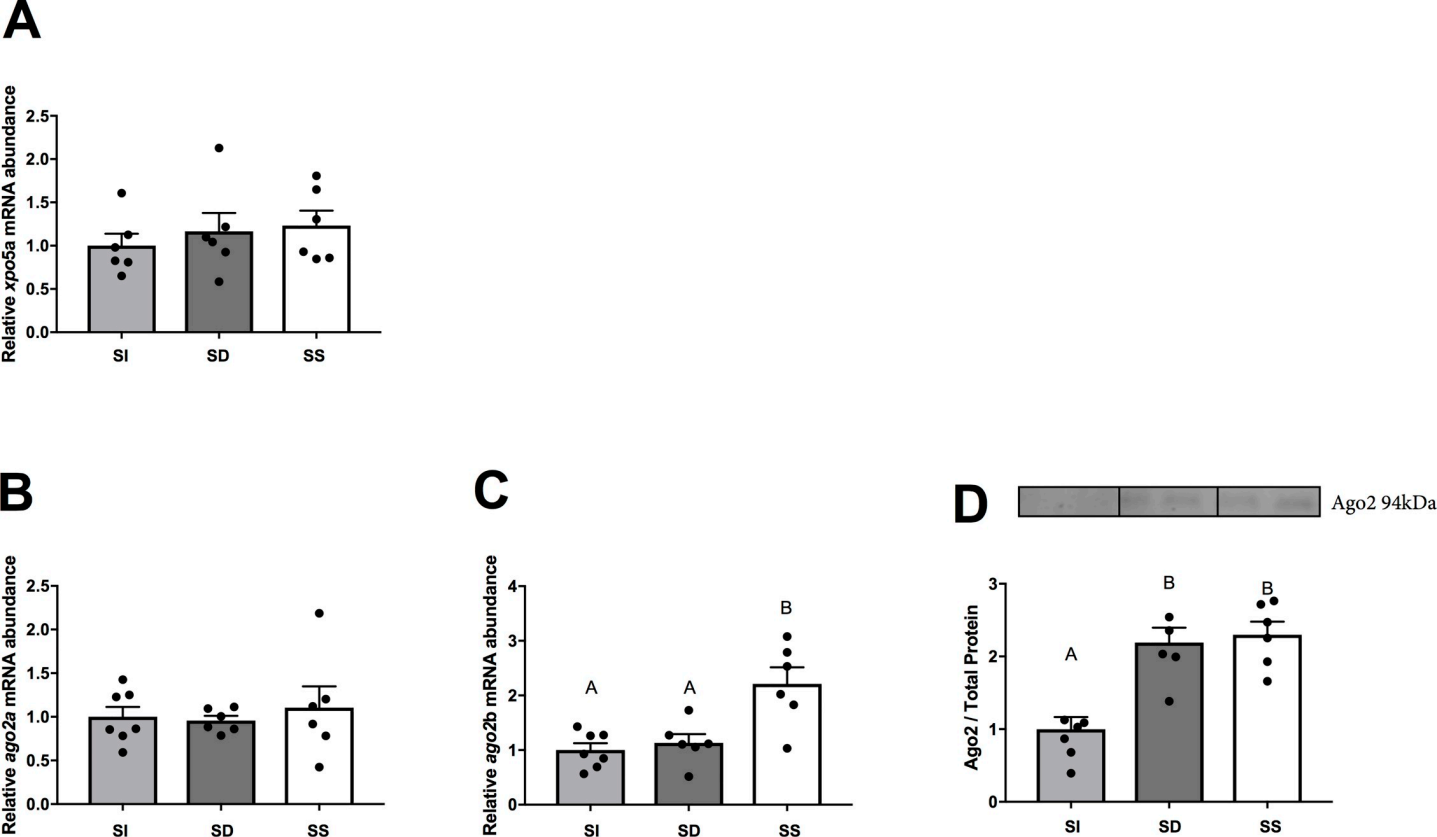
Relative steady-state mRNA abundance (+S.E.M.) of hepatic canonical miRNA biogenesis components exportin 5a (**A**), and argonaute 2a and argonaute 2b (**B-C**), as well as representative bands of argonaute protein abundance (**D**) in SI, SD and SS rainbow trout, *Oncorhynchus mykiss*. The mRNA abundance data were initially normalized using the Normagene approach, and protein data were initially normalized to total protein. Both mRNA abundance and protein data were then normalized to the SI group values to visualize relative fold-changes in SS and SD trout. Filled circles representing individual datapoints are additionally plotted for each group. A one-way ANOVAs followed by Tukey’s test was used for analysis. A p-value of p < 0.05 was used as cut-off for significant effects.

Responsiveness of the hepatic canonical miRNA biogenesis pathway to social interaction was probed at the mRNA abundance and protein levels in SI, SD, and SS rainbow trout (**Fig 2**). Specifically, transcript abundances of the two salmonid paralogues of exportin 5, *xpo5a* (**Fig 2A**) and argonaute 2, *ago2a* and *ago2b* (**Fig 2B and 2C**), were quantified, and Ago2 was also assessed at the protein level (**Fig 2D)**. The hepatic mRNA abundance of *xpo5a* (F_2,15_ = 0.24, p = 0.7885; **Fig 2A)** did not change significantly compared to the SI in SD and SS fish, but *xpo5b* abundance was too low to be reliably quantified. Although the hepatic abundance of *ago2a* mRNA did not change significantly among groups (F_2,16_ = 0.22, p = 0.2215; **Fig 2B**), significant changes were observed in *ago2b* (F_2,16_ = 10.58, p = 0.0012; **Fig 2C**), which increased significantly in SS trout compared to SD or SI trout (p < 0.05). At the protein level, Ago2 quantification (F_2,16_ = 16.13, p = 0.0001; **Fig 2D**) revealed significant increases between SI and both SD and SS groups (p < 0.05 in both cases).

### 3.2. Small RNA next generation sequencing and identification of social status dependent regulation of hepatic miRNAs in rainbow trout

**Fig 3:**
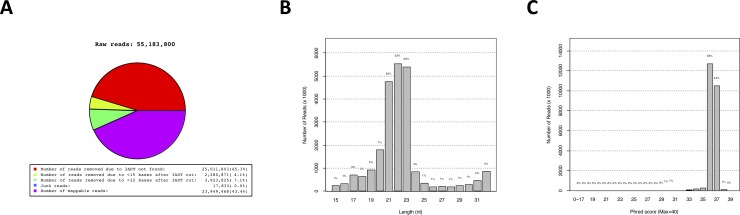
Number of raw reads, removed reads and mappable reads used in the small RNA next generation sequencing analysis (**A**), length distribution of reads (**B**), and Phred Score (**C**), an indicator of base call accuracy. A Phred score > 30 indicates 99.9% accuracy, or a 1:1000 probability of a false base call.

Of the overall ~55.18 M raw reads, 36.26 M reads (56.5%) were excluded firstly owing to a lack of 3’adaptor or 3’ADT (~25.01 M or 45.3%), and secondly because of nucleotide size outside the targeted range of 15–32 nt (~6.20 M or 11.2%) after 3’ADT sequence screening, resulting in ~40.60 M mappable reads (**Fig 3A**), of which 78% exhibited a size between 19 and 23 nt (**Fig 3B**). The Phred Score distribution of reads was larger than 30 (**Fig 3C**), indicating a probability of incorrect base calls of less than 1 in 1000 reads (or 99.9% accuracy). With regard to individual sample sequencing depth, a range between ~2.42 M and ~6.06 M mappable reads per sample was obtained (**S3 Table**). Following quality assurance of small RNA sequencing, the total of ~23.95 M mappable reads (**Fig 3A**) was annotated into specific groups according to the described pipeline (**S1 Table)**. In this process, ~14.73 M or 61.5% of reads were annotated as 591 known miRNAs (Group 1), whereas ~2.42 M or 10.1% of reads were annotated as 983 predicted miRNAs (Groups 2–4). A total of ~7.53 M or 31.4% of mappable reads was annotated as other RNA species or did not yield a hit (**Table 3** and **Table 4**). Thus, we identified a total of 1574 unique miRNAs, the specific sequences of which are available in **S2 Table**.

**Table 3:** Number of annotated small RNA sequences by group as defined in the bioinformatics analysis pipeline flow chart in Fig 2.

Group	# Sequences	% Mappable Sequences
Raw	55,183,800	
Total mappable reads	23,949,468	100
Group 1a	9,747,348	40.7
Group 1b	4,977,912	20.8
Group 2a	235,217	1
Group 2b	92,526	0.4
Group 3a	3,393	0
Group 3b	4,573	0
Group 4a	32,125	0.1
Group 4b	1,844,196	8.6
Mapped to mRNA	3,374,845	7.7
Mapped to other RNAs (RFam: rRNA, tRNA, snRNA, snoRNA and others)	3,380,212	14.1
Mapped to Repbase	165,394	0.7
Nohit	2,141,579	8.9

**Table 4:** Number of annotated mature miRNA sequences by group as defined in the flow chart depicted in Fig 2.

miRNAs	Group	# Unique miRs
**Known miRs**		
of specific species^1^	Group 1a	150
of selected species, but novel to specific species^2^	Group 1b	441
**Predicted miRs**		
Mapped to known miRs of selected species and genome; with hairpins	Group 2a	251
Mapped to known miRs of selected species and genome; no hairpins	Group 2b	130
Mapped to known miRs and miRs of selected species but not mapped to genome	Group 3a	83
Mapped to known miRs of selected species, but not mapped to genome	Group 3b	313
Not mapped to known miRs but mapped to genome and with hairpins	Group 4a	582
Overall (Unique miRs)		1574

**Fig 4:**
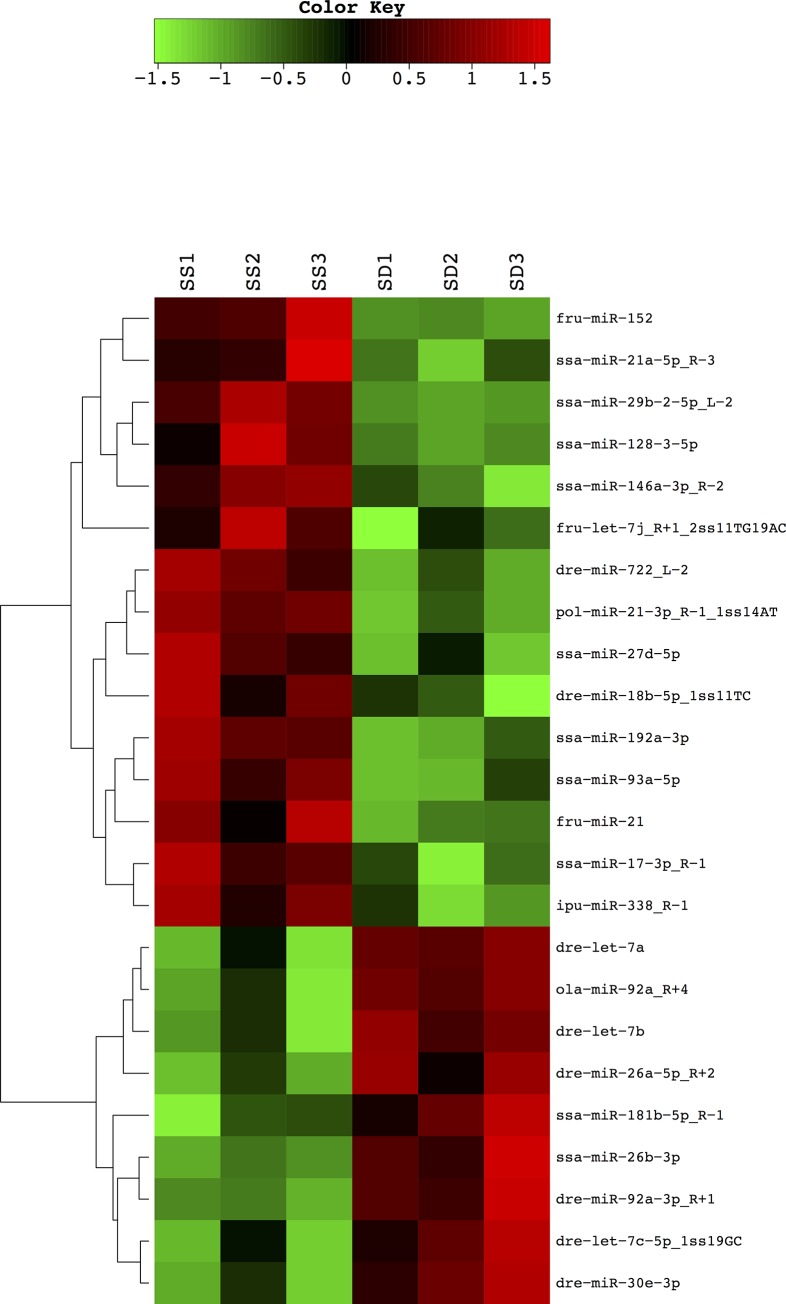
Heatmap showing hierarchical clustering of differentially regulated hepatic miRNAs between SS (SUB1-3) and SD (DOM1-3) rainbow trout, analyzed by t-tests. See text for explanation.

**Fig 5:**
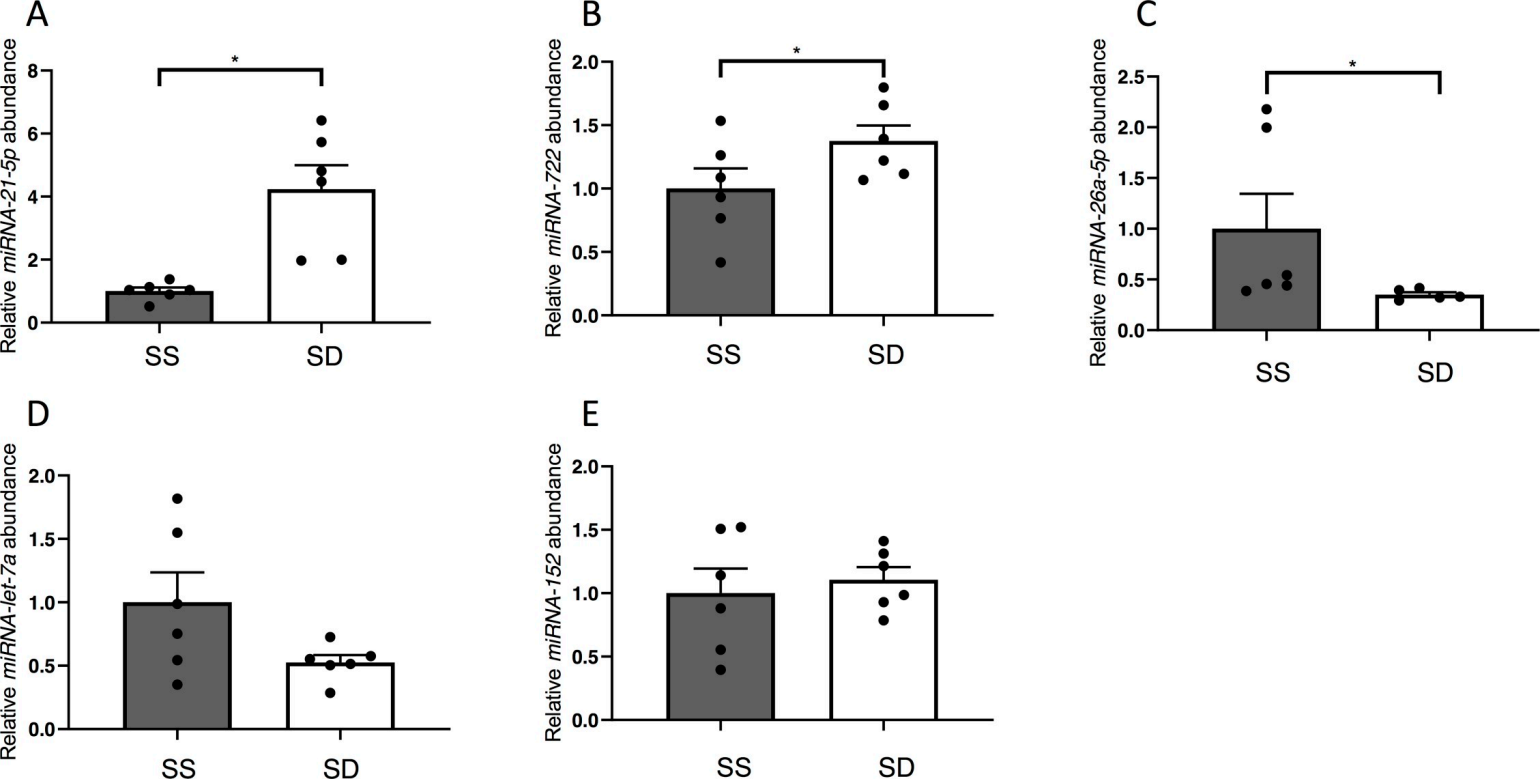
Relative steady-state abundance (+S.E.M.) of the most abundant differentially regulated hepatic miRNA, *miRNA-21-5p*, as well as *miRNA-722*, *miRNA-26a-5p*, *miRNA-let-7a* and *miRNA-152* in liver of SD and SS rainbow trout, *Oncorhynchus mykiss*. A one-tailed Welch’s t-test was used for analysis, and a p-value of p<0.05 was used as cut-off for significant effects.

Analysis by t-tests (p < 0.05), revealed a total of 24 differentially expressed miRNAs between SD and SS trout, with 9 miRNAs that were expressed more abundantly in the liver of SD rainbow trout, and, conversely, 15 miRNAs that exhibited significantly higher abundance in SS trout (**Fig 4**). A detailed list of the differential expression analysis can be found in **S3 Table**. To validate miRNA abundance identified by small RNA next generation sequencing, we quantified the most abundant differentially regulated miRNA, *miRNA-21-5p* (**S3 Table**), by a Taqman based real-time RT-PCR, and found significantly higher hepatic *miRNA-21-5p* abundance (One-tailed Welch’s t test: df = 5.229, t = 4.184, p = 0.0039, **Fig 5A**) in SS rainbow trout compared to SD animals. Additionally, we measured relative transcript abundance of *miRNA-722*, *miRNA-26a-5p*, *miRNA-let-7a*, and *miRNA-152* (**Fig 5B–5E**). Similarly, to *miRNA-21-5p*, we found significantly increased expression of *miRNA-*722 in SSs relative to SD fish (One-tailed Welch’s t test: df = 9.363, t = 1.873, p = 0.0463, **Fig 5B**). Further validating the NGS data, *miRNA-26a-5p* (One-tailed Welch’s t test: df = 7.203, t = 2.959, p = 0.0102, **Fig 5C**) and *miRNA-let-7a* (One-tailed Welch’s t test: df = 5.608, t = 1.953, p = 0.0510 **Fig 5D**) demonstrated significantly and marginally significantly lower expression respectively in SS fish relative to SD fish. *miRNA-152* (One-tailed Welch’s t test: df = 7.451, t = 0.4882, p = 0.3197 **Fig 5E**) did not show a significant difference in relative miRNA abundance between SS and SD rainbow trout.

### 3.3. In silico analysis of differentially regulated miRNA targets predicts glucose, lipid, and protein metabolic pathways as being post transcriptionally regulated

**Fig 6:**
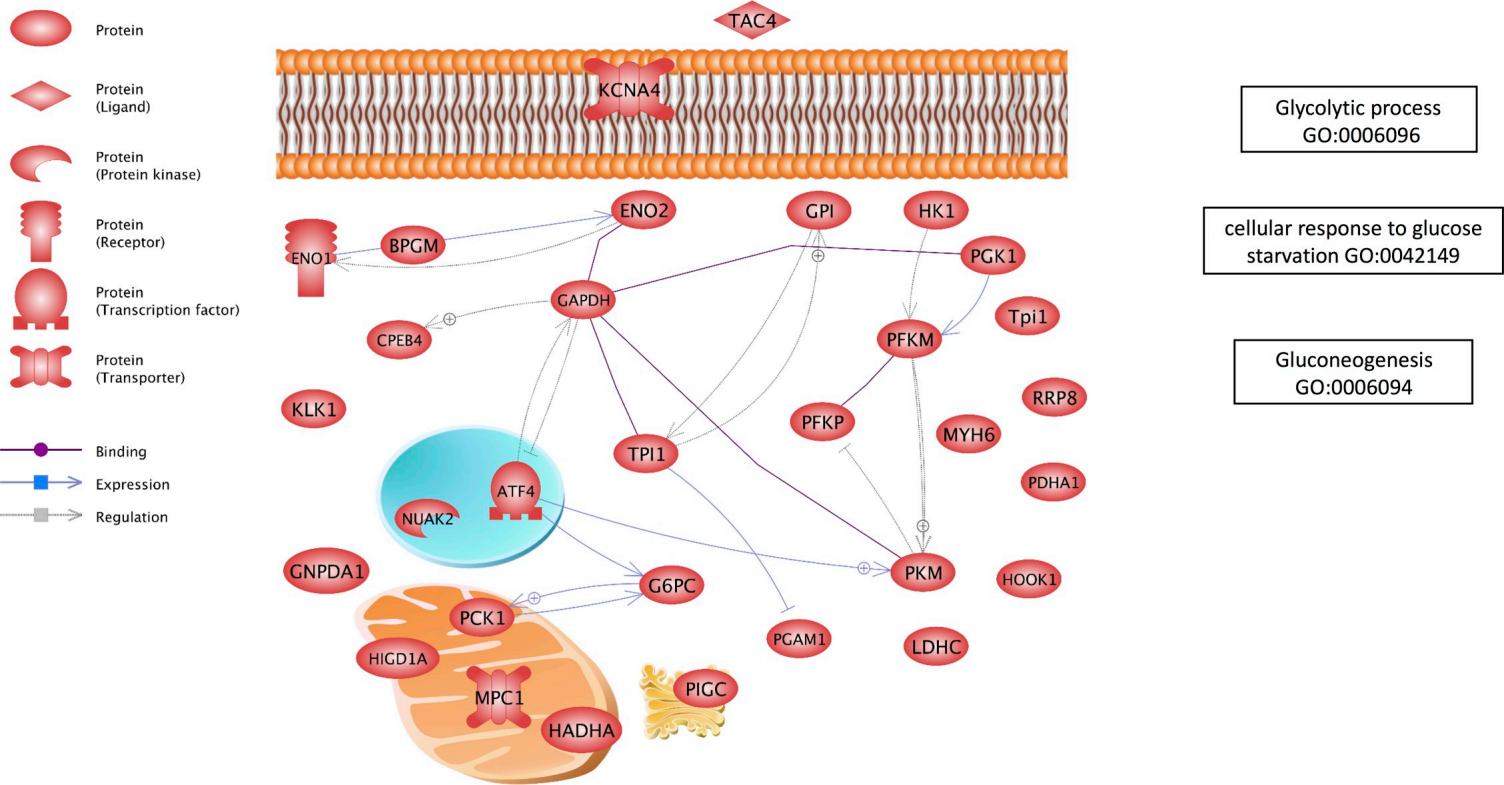
A gene network schematic depicting key predicted targets for miRNAs for gene ontology terms related to the theme of glucose regulation. Pathways were constructed in Pathway Studio. Each target in the pathway is predicted to be regulated by a hepatic miRNA that is differentially regulated between SS and SD rainbow trout.

To gain initial insight into genome-wide consequences of differentially regulated hepatic miRNAs between SS and SD rainbow trout, targets were predicted based on their theoretical 3’UTR capacity to bind the 24 differentially regulated miRNAs (**S4 Table**). Based on annotated GO terms of the targets compared to whole genome GO terms, we identified several pathways that were predicted to be regulated by these differentially regulated miRNAs (**S5 Table**). The pathways most relevant to the differential regulation of hepatic metabolic pathways were related to glucose [4, 6], lipid [7] and to some extent, protein metabolism [7] in SD and SS rainbow trout liver. Additionally, pathways related to mitochondrial dynamics were predicted to be targeted by differentially regulated miRNAs between SS and SD fish. Results of the analysis are summarized in **Table 5**. In the case of glucose metabolism, we visualized individual genes and pathways predicted to be post-transcriptionally regulated by differentially regulated miRNAs using sub-network enrichment (SNEA) analysis (**Fig 6, S1 Fig**).

**Table 5:** Significant metabolism-related GO-term enrichment of *in silico* predicted target mRNAs of miRNAs that were differentially regulated between dominant and subordinate rainbow trout. The full list of enriched GO terms can be consulted in **S4 Table**.

Targeted metabolic pathway or process	GO term	Fisher raw p-value
**General intermediary metabolism**		0.036
	GO:0097009 energy homeostasis	
**Glucose metabolism**		
	GO:0042149 cellular response to glucose starvation	0.019
	GO:0006094 gluconeogenesis	0.005
	GO:0006108 malate metabolic process	0.001
	GO:0004471 malate dehydrogenase (decarboxylating) (NAD+) activity	0.006
	GO:0006096 glycolytic process	0.031
	GO:0044262 cellular carbohydrate metabolic process	0.035
**Lipid metabolism**		
	GO:0034379 very-low-density lipoprotein particle assembly	0.001
**Protein/AA metabolism**		
	GO:0002181 cytoplasmic translation	0.001
	GO:0042274 ribosomal small subunit biogenesis	0.006
	GO:0006415 translational termination	0.009
	GO:0006622 protein targeting to lysosome	0.045
**Mitochondria**		
	GO:0008053 mitochondrial fusion	0.014
	GO:0042407 cristae formation	0.006
	GO:0060263 regulation of respiratory burst	0.006
	GO:0009055 electron carrier activity	0.009
	GO:0070469 respiratory chain	0.010
	GO:0008121 ubiquinol-cytochrome-c reductase activity	0.001
	GO:0005744 mitochondrial inner membrane presequence translocase complex	0.017

### 3.4. SS status induces *pck1* transcript in rainbow trout liver

**Fig 7:**
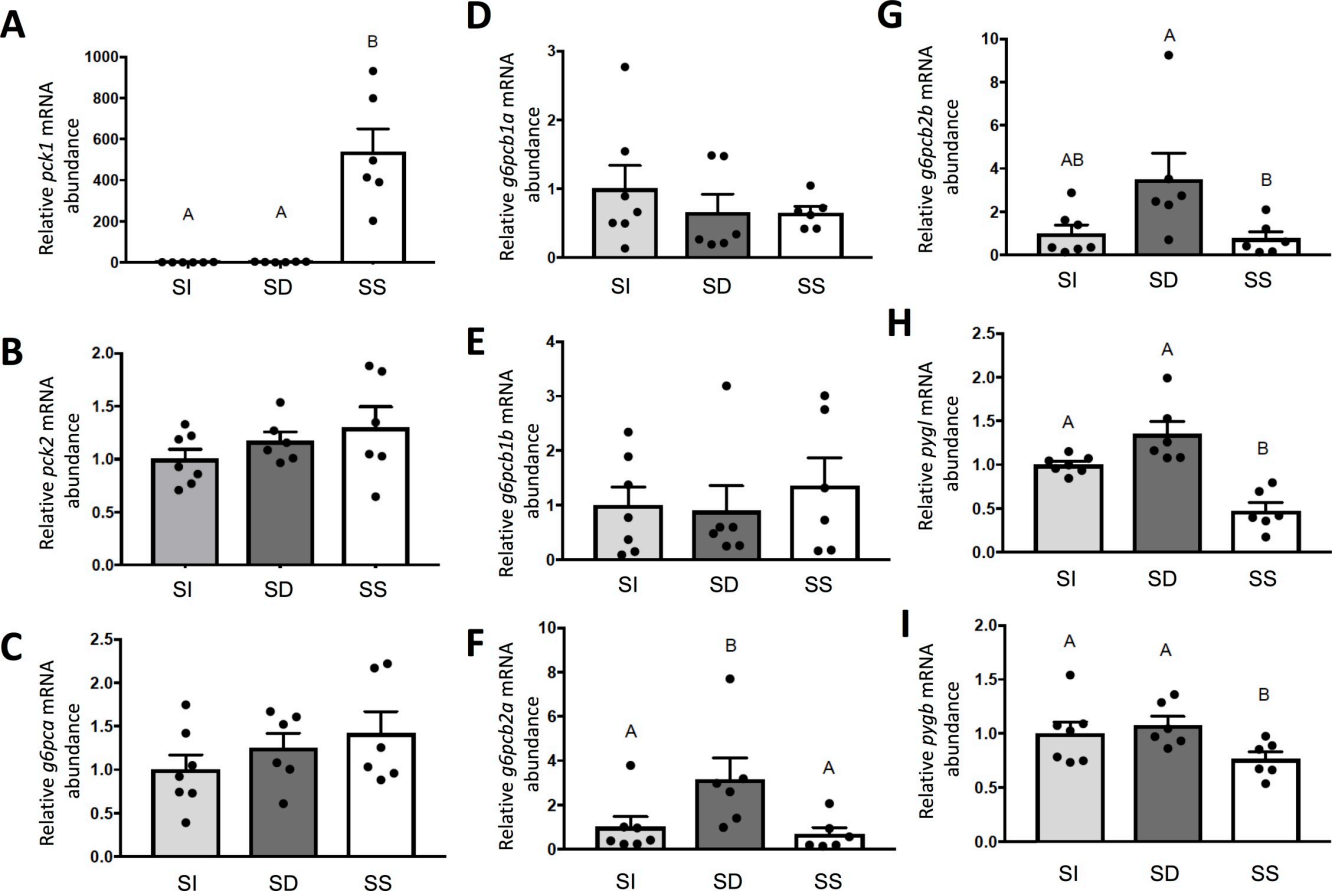
Steady state mRNA abundance (+S.E.M.) of genes involved in hepatic glucose metabolism, including gluconeogenic enzyme isoforms cytoplasmic phosphoenolpyruvate carboxykinase *pck1* (**A**), mitochondrial phosphoenolpyruvate carboxykinase *pck2* (**B**), gluconeogenic enzyme paralogues of glucose-6-phosphatase (**C-G**), and liver (**H**) and brain (**I)** isoforms of the glycogenolytic enzyme, glycogen phosphorylase. Data for SI, SD and SS rainbow trout (*Oncorhynchus muykiss*) were normalized using the Normagene algorithm, and then expressed relative to values for SI fish. A one-way ANOVAs followed by Tukey’s post-hoc was used for analysis. A p-value of p<0.05 was used as cut-off for significant effects.

Given the enrichment of intermediary metabolic pathways predicted to be post-transcriptionally regulated by differentially regulated miRNAs in SD and SS rainbow trout liver, and the well-described effects of social status on hepatic glucose metabolism, particularly gluconeogenesis and glycogen metabolism, we quantified mRNA abundances of gluconeogenic enzyme paralogues of phosphoenolcarboxykinase (*pck1* and *pck2*, **Fig 7A and 7B**), glucose-6-phosphatase (*g6pca*, *g6pcb1a*, *g6pcb1b*, *g6pcb2a*, and *g6pcb2b*, **Fig 7C–7G**), and glycogen phosphorylase (liver *pygl* and brain *pygb* forms, **Fig 7H and 7I)**. The mRNA abundance of cytoplasmic *pck1* (F_2,15_ = 132.10, p = 0.0001), but not mitochondrial *pck2* (F_2,16_ = 2.84, p = 0.0880), was significantly higher in SS trout compared to both SI and SD rainbow trout (p < 0.0001). No changes were found in mRNA expression of *g6cpca* (F_2,16_ = 1.04, p = 0.9414), *g6pcb1a* (F_2,16_ = 0.7042, p = 0.5092) and *g6pcb1b* (F_2,16_ = 0.2664, p = 0.7694).However, hepatic *g6pcb2a* (F_2,16_ = 6.091, p = 0.0108) and *g6pc2b* (F_2,16_ = 4.904, p = 0.0218) mRNA abundances were significantly higher in SD trout compared to SS trout (p = 0.0121 and p = 0.0290, respectively) and significantly or marginally higher than in SI trout (p = 0.0391 and p = 0.0502, respectively). The hepatic mRNA abundances of both *pygl* (F_2,16_ = 18.59, p = 0.0001) and *pygb* (F_2,16_ = 3.695, p = 0.0480) were lower in SS trout compared to SD trout (p = 0.0001 and p = 0.0469, respectively). In the case of *pygl*, the hepatic mRNA abundance in SS was also significantly reduced compared to SI animals (p = 0.0036).

### 3.5. Lipolytic and mitochondrial fission markers are elevated in SS rainbow trout

**Fig 8:**
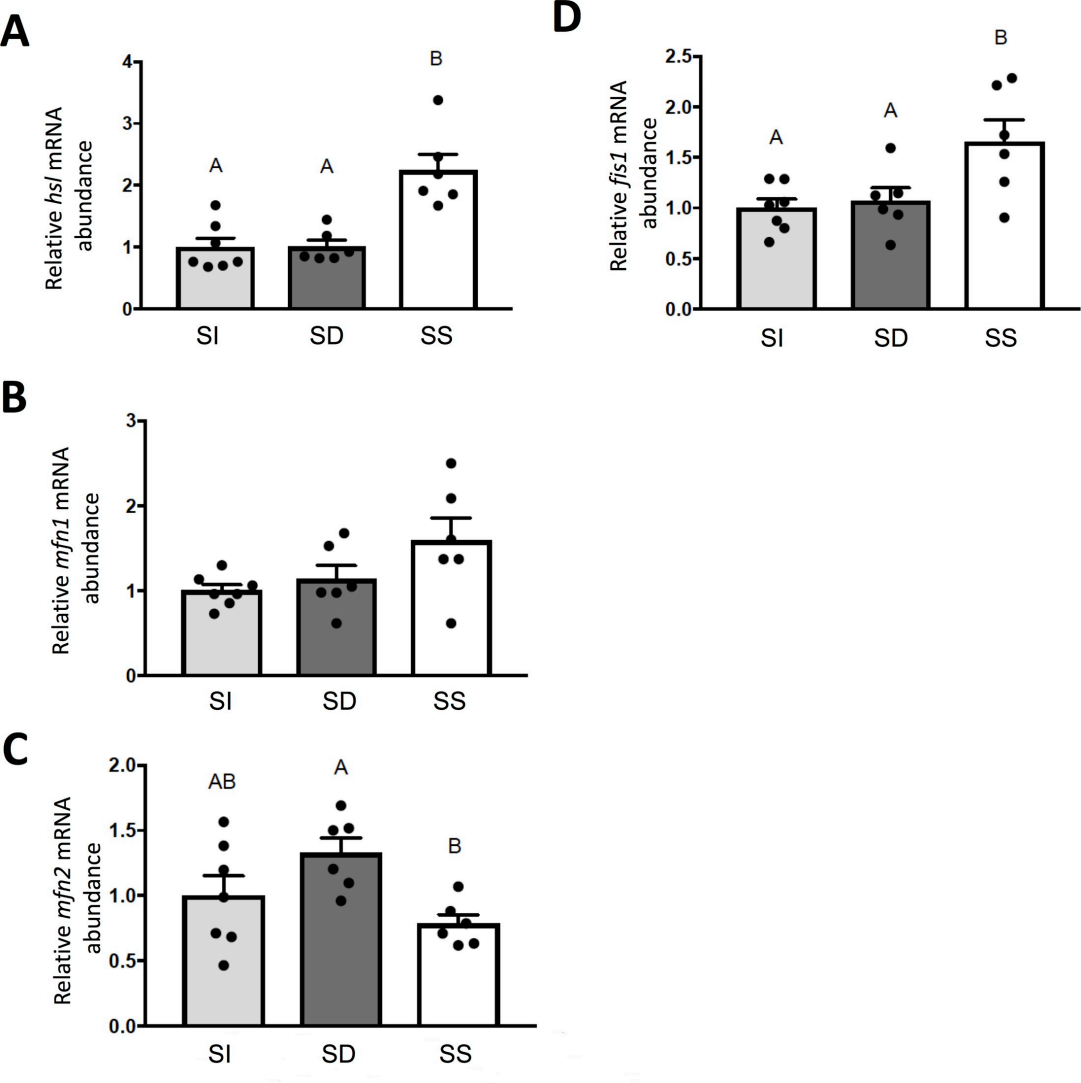
Steady state mRNA abundance (+S.E.M.) of genes involved in hepatic lipid metabolism, hormone sensitive lipase, *hsl* (**A**), and those involved in the regulation of mitochondrial dynamics including mitofusin 1, *mfn1* (**B**) mitofusin2, *mfn2* (**C**) and mitochondrial fission protein, *fis1* (D). Data for SI, SD and SS rainbow trout (*Oncorhynchus mykiss*) were normalized using the Normagene algorithm, and then expressed relative to values for SI fish. A one-way ANOVAs followed by Tukey’s post-hoc was used for analysis. A p-value of p<0.05 was used as cut-off for significant effects.

Hepatic expression of hormone sensitive lipase *hsl* (F_2,16_ = 18.59, p = 0.0001, **Fig 8A**) was significantly higher in SS fish compared to SD (p = 0.005) and SI fish (p = 0.001). With regard to hepatic mRNA abundance of mitochondrial fusion proteins, *mfn1* (F_2,16_ = 2,095 p = 0.1556, **Fig 8B**) did not differ among treatment groups, whereas *mfn2* (F_2,16_ = 4.753, p = 0.0240, **Fig 8C**) was lower in SS trout compared to SD trout (p = 0.0194). Conversely, hepatic mRNA abundance of the mitochondrial fission protein, *fis1* (F_2,16_ = 5.606, p = 0.0143, **Fig 8D**) was higher in SS trout compared to SD (p = 0.0417) and SI trout (p = 0.0175).

### 3.6. SS trout exhibit significantly increased circulating glucose concentration

**Fig 9:**
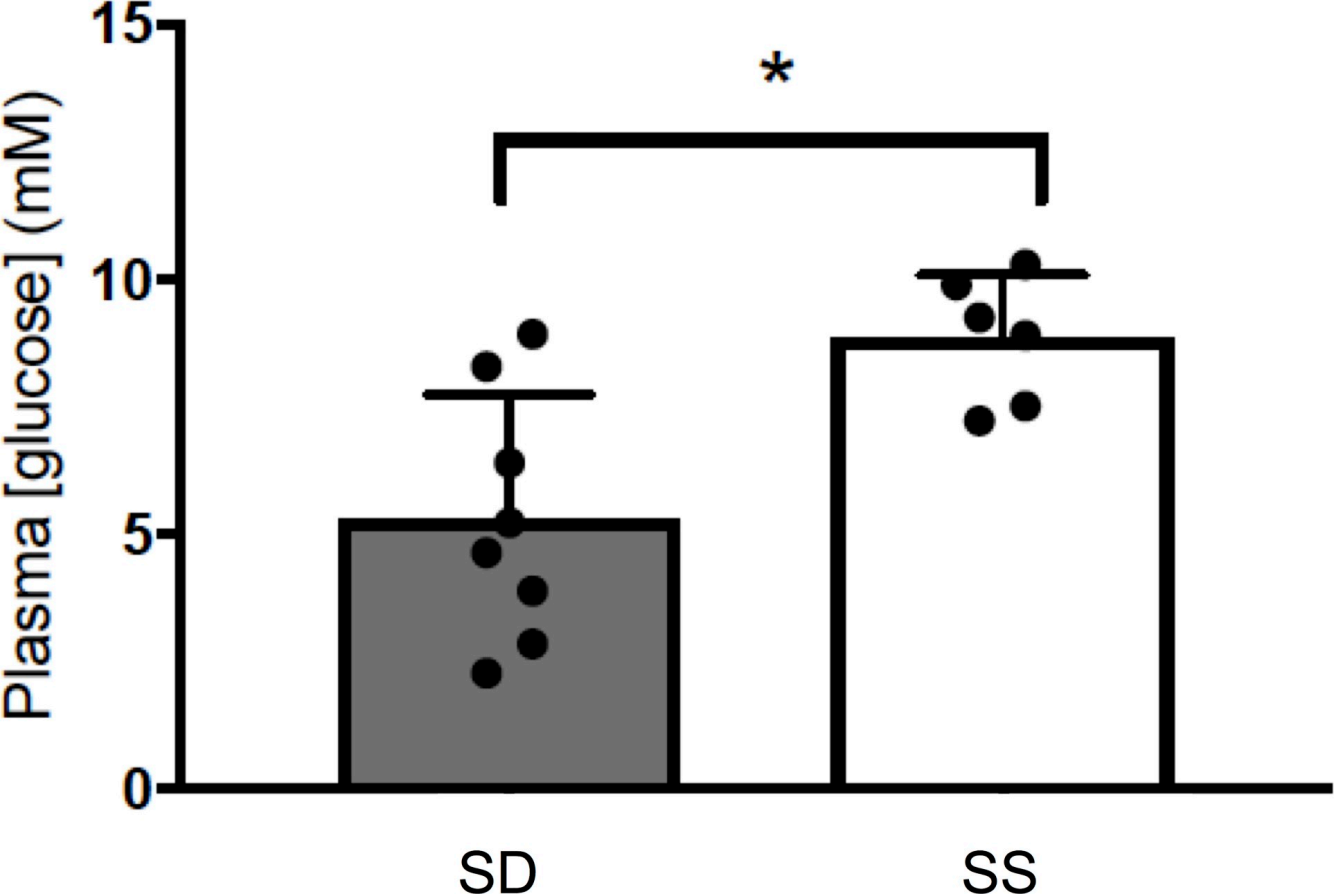
Circulating glucose concentrations in SD and SS rainbow trout (*Oncorhynchus mykiss*). Data were analyzed using Welch’s t-test. A p<0.05 was used as cut-off for significant effect.

To completely assess the SS phenotype at the metabolite level, we quantified circulating glucose concentrations to complement previously reported plasma lipid metabolite changes, specifically increases in triglyceride in SD rainbow trout and increases in free fatty acids in SS rainbow trout (7). SS trout exhibited significantly higher circulating glucose concentrations than SD rainbow trout (t_12_ = 3.251, p < 0.01; **Fig 9**).

### 3.7. miRNA-target gene relationships

Using correlative analysis of miRNA and predicted target gene expression, we identified several miRNA-mRNA expression pairs exhibiting significant correlation coefficients (**Table 6**). With regard to glucose metabolism, significant negative correlations between *miR-27d-5p* and *pck1*, and *miR-21-3p* and *pygb* were detected. With regard to lipid metabolism, the majority of miRNAs predicted to target *hsl* exhibited positive correlations with the *hsl* transcript, which was significant in the case of *miR-21-3p*. Exclusively negative relationships were identified for miRNAs predicted to target mitofusins, reaching significance for *miR-18b-5p*. Conversely, *miRNA-21-3p* and its predicted target *fis1* exhibit a significant positive correlation.

**Table 6:** Correlation of expression of differentially regulated miRNAs and predicted targets involved in pathways relevant to hepatic regulation of glucose metabolism.

Metabolic pathway and target gene	miRNA	Pearson correlation coefficient	Significance
**Glucose metabolism**			
*pck1* GSONMT00082468001	*ssa-miR-27d-5p*	-0.75	p<0.05
*pck2* GSONMT00059643001	*ssa-miR-128-3-5p*	0.19	n.s.
*g6pca* GSONMT00076843001	*ipu-miR-338_R-1*	-0.14	n.s.
*g6pcb1*.*a* GSONMT00076841001	*none*	none	none
*g6pcb1*.*b* GSNOMT00066036001	*ssa-miR-181b-5p_R-1*	0.13	n.s.
*g6pcb2*.*a* GSNOMT00013076001	*dre-miR-92a-3p_R+1*	0.62	n.s.
*g6pcb2*.*b* GSNOMT00014864001	*none*	none	n.s.
*pygb* GSNOMT00009821001	*pol-miR-21-3p_R-1_1ss14AT*	-0.84	p<0.05
**Lipid metabolism**			
*hsl* GSNOMT00023441001	*fru-miR-21*	0.58	n.s.
*hsl* GSNOMT00023441001	*pol-miR-21-3p_R-1_1ss14AT*	0.83	p<0.05
*hsl* GSNOMT00023441001	*fru-miR-152*	0.71	n.s.
*hsl* GSNOMT00023441001	*ssa-miR-93a-5p*	0.69	n.s.
*hsl* GSNOMT00023441001	*dre-miR-18b-5p_1ss11TC*	0.70	n.s.
*hsl* GSNOMT00023441001	*ipu-miR-338_R-1*	0.61	n.s.
*hsl* GSNOMT00023441001	*dre-miR-30e-3p*	-0.71	n.s.
**Mitochondrial dynamics**			
*mfn1* GSNOMT00027395001	*dre-miR-let-7a*	-0.71	n.s.
*mfn1* GSNOMT00027395001	*dre-miR-let7c-5p_1ss19GC*	-0.62	n.s.
*mfn1* GSNOMT00027395001	*ssa-miR-27d-5p*	-0.62	n.s.
*mfn2* GSNOMT00004526001	*dre-miR-18b-5p_1ss11TC*	-0.75	p<0.05
*mfn2* GSNOMT00004526001	*fru-miR-152*	-0.44	n.s.
*mfn2* GSNOMT00004526001	*pol-miR-21-3p_R-1_1ss14AT*	-0.73	n.s.
*mfn2* GSNOMT00004526001	*ssa-miR-29b-2-5p_L-2*	-0.61	n.s.
*fis1* GSNOMT00039888001	*pol-miR-21-3p_R-1_1ss14AT*	0.75	p<0.05

## 4. Discussion

The current study reveals that the hepatic miRNA biogenesis pathway is induced in interacting SD and SS juvenile rainbow trout compared to SI-treated trout that did not experience social interactions. In addition to the previously reported increase in *drosha* mRNA abundance [7], which is responsible for a crucial step in miRNA biogenesis [16, 32], we here report the elevation of Ago2 protein abundance in both SS and SD rainbow trout compared to individually housed SI trout, further supporting the notion of increased hepatic miRNA biogenesis and function in response to agonistic social interactions indicative of an enhanced functional role for miRNAs in post-transcriptional regulation of hepatic gene expression in socially antagonistic conditions.

To begin to delineate qualitative consequences of hepatic miRNA expression between SS and SD rainbow trout, we utilized a small miRNA next generation sequencing approach to identify 24 differentially regulated miRNAs, with 15 that exhibited increased expression, and 9 miRNAs that exhibited decreased expression in the liver of SS trout compared to SD trout. Differences in miRNA expression with social status may be mediated by differential biogenesis and/or turnover mechanisms [14,32]. While our experiment does not allow to resolve the relative contribution of each mechanism, the previously reported increase in *drosha* mRNA abundance in both SS and SD rainbow trout compared to individually housed SI controls [7] is indicative of at least a partial contribution of biogenesis to this differential expression. In order to explore the possible functional relevance of differentially expressed miRNAs, we used a genome-wide *in silico* approach [21] to predict specific targets and their enrichment by GO-term function. Because miRNAs play important roles in the regulation of (hepatic) energy metabolism not only in mammals (13,31), but also in fish, and in trout in particular [14,15] and social status-dependent regulation of hepatic glucose [4,6], lipid [7], and possibly protein metabolism [7] has been well described in SD versus SS rainbow trout, we focused our analysis of pathways predicted to be targeted by differentially regulated miRNAs on those relevant to hepatic glucose production, especially gluconeogenesis and glycogenolysis, lipolytic pathways, and pathways involved in protein synthesis. Additionally, pathways related to mitochondrial dynamics were examined because they were identified as being enriched targets of differentially regulated miRNAs in our *in silico* analysis, and have recently been linked to differential fuel utilization in response to nutrient availability and stress, at least in mammals [33–36].

With regard to gluconeogenesis target genes, we identified a very strong induction of *pck1*, the cytosolic form of phosphoenolpyruvate in SS fish, with no changes in the reported mitochondrial form of the enzyme, *pck2*. These results strongly suggest that the previously observed significant increase in global hepatic Pck activity in SS trout [4] is regulated at least in part, at the transcriptional and/or post-transcriptional level of the *pck1* gene. It is well documented that the SS trout phenotype is characterized by chronically elevated cortisol, as well as short term fasting [7], and both factors have been shown to contribute to increased *pck1* transcription in mammals. Indeed, cytosolic *pck1* is considered to be the principal target of endocrine and nutritional regulation [37]. This finding is in line with earlier studies investigating unspecified *pck* mRNA abundance before the publication of the rainbow trout genome facilitated paralogue specific *pck* transcript amplification. Our study clearly assigns this transcriptional response to the cytosolic *pck1* in rainbow trout, and the ability to distinguish between paralogues may have contributed to the much stronger fold-induction compared to pre-genome studies [38]. Additionally, the higher fold-induction may be explained by the fact that the current study investigated SS fish, which in addition to chronically elevated cortisol concentrations experienced a *de facto* short-term fast because of the monopolization of food by SD fish in the dyad [7]. Since it is well-described that cortisol interacts synergistically with glucagon in the regulation of *pck1* transcript abundance [37] future studies should investigate the role of fasting, glucagon and cortisol on the hepatic *pck1* transcript. Such synergism may also explain why both higher fold *pck1* mRNA abundance and global Pck activity are observed in SS trout, while cortisol injected trout reveal increased *pck* mRNA abundance but not activity [39]. Within the gluconeogenic pathway, we additionally probed *g6pc* paralogues and found no change in mRNA abundance of *g6pca* and *g6pcb1* paralogues. Unexpectedly, we identified a paradoxical increase in *g6pcb2* paralogues in SD fish, confirming an atypical regulation of these recently described novel members of the teleost *g6pc* family [40]. Because overall circulating glucose concentration was increased in SS fish compared to SD fish in our current study (**Fig 9)**, our data suggest a functional contribution of gluconeogenesis, likely via transcriptional regulation of *pck1* in particular, to this phenotype. We found that *g6pcb2b* mRNA abundance is increased in SD, normoglycemic trout that monopolize food during social interactions, thereby acquiring a double ration. A similar, differential regulation of *g6pcb2b* paralogue mRNA abundance was reported previously in response to refeeding, especially with diets rich in carbohydrates [40]. With regard to glycogen phosphorylase paralogues, we identified lowered hepatic mRNA abundance of the liver and brain isoforms, *pygl* and *pygb*, in SS rainbow trout. SS rainbow trout exhibit significantly reduced hepatic glycogen content compared to both control and SD fish, with SS fish reaching concentrations of as low as 16% of hepatic glycogen content measured in SD fish [6]. This coincides with significant reductions in basal hepatic Gp activity, which is, however, likely limited by the decreased amount of glycogen, as total GP activity and GPα specific activity (assessed by adding additional glycogen or glycogen and caffeine, respectively) is lower in SSs compared to controls, but exhibits the lowest values in SD fish [6]. This reported lowered GP activity compared to control fish has been interpreted to protect already depleted hepatic glycogen storage in SS fish from further glycogenolysis in the face of low food intake and chronic stress, and our current results indicate that this process in SS fish is, at least in part, regulated at the mRNA abundance level of the hepatically expressed paralogues *pygl* and *pygb*. Conversely, the reported lowest total and GPα activity in SD trout interpreted to be reflective of rapid restocking of hepatic glycogen reserves following the initial acute stress of social hierarchy establishment do not appear to be linked to transcriptional regulation of these transcripts, as indicated by similar transcript abundances between control and SD fish. It is possible that chronically elevated cortisol levels are responsible for a mRNA based regulation as opposed to well described posttranslational regulation of glycogen phosphorylase activities. Indeed, conflicting results of cortisol on hepatic glycogen in rainbow trout have been reported [39], likely pointing to different baseline conditions, duration of the cortisol signaling and/or interaction between different regulating factors as mechanisms in the regulation of hepatic glycogen content in response to cortisol.

In addition to glucose metabolism, lipid metabolism exhibits pronounced differences between SD and SS rainbow trout [7]. Increased circulating triglycerides in SD rainbow trout correlate with increased hepatic indices of *de novo* lipogenesis, whereas elevated circulating free fatty acids correlate with indices of fatty acid β-oxidation in SS rainbow trout [7]. These results suggest that SD trout, which monopolize food use the energetically costly process of *de novo* lipogenesis to contribute to storage of lipid reserves, whereas SS fish rely on lipolytic processes to fuel metabolism, including hepatic metabolism [7]. Here, we report a significant increase in hepatic mRNA abundance of a paralogue of hormone sensitive lipase (*hsl*), providing further evidence for lipolytic activity in SS fish. In rainbow trout, hepatic *hsl* has been shown to be strongly regulated by nutritional and endocrine stimuli [41–42], and our finding of *hsl* upregulation in SS fish, which experience little or no food intake during social interactions, is consistent with data previously reported for *hsl* paralogues observed in trout in a comparable fasted state [41–42].

Because pathways linked to mitochondrial dynamics were predicted as being targeted by miRNA and are, at least in mammals, regulated by stress and nutritional status and functionally linked to oxidative glucose and lipid metabolism [33–36], we profiled markers of mitochondrial fusion and fission. We identified a decrease in mRNA abundance of *mfn2* in the liver of SS fish and, conversely, an increase in *fis1* transcript abundance, consistent with the opposing regulation often observed in these markers to coordinate mitochondrial dynamics [34]. Cortisol, the end-product of the stress axis, has been shown to increase mitochondrial fission, which, in turn, is functionally linked to increased gluconeogenesis in hepatoma cells [35]. Interestingly, liver-specific ablation of *mfn2* also contributes to increased gluconeogenesis [36], suggesting that in our model system, glucose liberation in SS fish may, at least in part, also mediated at the level of mitochondrial dynamics. Functional studies at the cellular level clearly are needed to support these gene expression findings.

Collectively, our findings at the gene expression level for paralogues involved in glucose metabolism, lipid metabolism and mitochondrial dynamics are consistent with previously reported metabolic changes [4,6,7], and suggest an important role for transcriptional and/or post-transcriptional regulation of gene expression in eliciting SD versus SS metabolic phenotypes. Because the specific transcript targets examined in this study were components of metabolic pathways identified as being regulated by differentially expressed miRNAs, and indeed predicted to be specifically targeted by differentially regulated miRNAs (**Table 5**), we investigated whether specific miRNA-mRNA target abundances were correlated (**Table 6**). Although significant correlations do not provide proof of functional interactions, the significant relationships in miRNA-target mRNA abundances that we observed prioritize these components for future functional studies.

A recent study has linked *miRNA-27* family members to the regulation of hepatic gluconeogenic enzymes, including PCK, suggesting that the predicted *miRNA-27d-5p*-*pck1* interaction in our study may be linked to evolutionarily deeply conserved roles of *miRNA-27* family members in the regulation of gluconeogenesis [43]. Through predictions and significant inverse relationships in abundance, we identified *miRNA-27d-5p*, *miRNA-21-3p* and *miRNA-18b-5p* as potential regulators of glucose liberation, lipolysis and mitochondrial dynamics. In rodent models, functional roles for *miR-21*-*3p* in hepatic carbohydrate and lipid metabolism in response to nutritional stress have been described [44]. However, these effects are mediated via different primary targets compared to those predicted in rainbow trout, likely reflecting the low degree of deeply conserved miRNA-mRNA relationships, which have undergone extensive rewiring between fish and mammals [45]. With regard to protein metabolism, our current study predicts a convergent regulation on different paralogues of S6 kinase, a translational regulator, by multiple miRNAs (*let-7a*, *let-7b*, *let-7c*, *miRNA-92a-3p*, *miRNA-181-5p*) that are downregulated in the liver of SS trout compared to SD trout (**S4 Table)**. This situation is suggestive of a post-transcriptionally-regulated increase in S6K. Consistent with this scenario, ribosomal S6 phosphorylation, an indicator of translational activity, was strongly induced in the liver of SS trout in our experimental animals [7], in line with the unexpected measurement of elevated rates of protein synthesis in the liver of SS rainbow trout (R.J. Saulnier, C. Best, D.J. Kostyniuk, K.M. Gilmour and S.G. Lamarre, unpublished results).

## 5. Conclusions, limitations and future perspectives

Overall, our study identifies increased protein abundance of Ago2 in dyad paired trout, suggesting that the miRNA pathway is important in the mediating posttranscriptional control of hepatic gene expression in both SS and SD rainbow trout. The nature of posttranscriptional regulation is differential between SS and SD rainbow trout, and differentially regulated miRNAs are predicted to target specific transcripts of important intermediary metabolic pathways of glucose, lipid and protein metabolism previously shown to be social status dependent in rainbow trout. Concerning the SI group, it is important to mention that a negative growth rate was observed, which, while not significantly different from growth rate observed in SD as is the case in SS, might be linked to catabolic reactions in this group, thereby affecting endpoints assessed in this group. Because the SS phenotype is associated with fasting, a chronic stress response and negative somatic growth, while the SD phenotype is associated with increased feed intake as it monopolizes food in dyads, the specific contribution of each of these factors to the observed hepatic miRNA regulation and their functional roles in mediating metabolic changes warrant further study.

Our framework therefore defines testable hypothesis for the involvement of specific miRNA-target mRNA pairs in the well-established metabolic differences in hepatic macronutrients metabolism, and identifies rainbow trout mitochondrial dynamics as a potential miRNA-regulated system that may contribute to differences in hepatic metabolism between SS and SD rainbow trout. Future studies are needed to functionally assess the role of specific miRNA-mRNA target pairs identified in our current study in the regulation of hepatic metabolism in rainbow trout. Specifically, the use of rainbow trout primary hepatocytes or cell lines transfected with plasmids containing 3’UTR and luciferase assays will allow to investigate physical interaction of miRNA and 3’UTR, while miRNA modulation approaches in the same systems allow to probe metabolic function, respectively (14). These assays are a necessary next step to validate identified candidates functionally, especially since *in silico* predictions suffer from false positives [25], and negative and positive correlations at a single sampling time only point towards possible modes of miRNA-mRNA interaction across time [46,47].

## Supporting information

S1 Table Flowchart representing the bioinformatics pipeline used to annotate and group miRNAs identified from small RNA next generation sequencing results. See text for explanation.
(XLSX)

S2 Table Next generation sequencing read summary, sequences and normalized counts. (XLSX)

S3 Table Differential hepatic miRNA expression between SS and SD trout (n = 3 per group) as determined by t-test. (XLSX)

S4 Table miRanda based annotated 3’UTR target prediction of differentially expressed miRNAs identified as being differentially expressed between SS and SD rainbow trout. Colours match heatmap designation in **Fig 4**, with green indicating predicted targets of miRNAs that have higher expression in SS rainbow trout liver, and red indicating predicted targets of miRNAs with higher expression in SD trout liver.
(XLSX)

S5 Table GO-term enrichment analysis of target genes predicted to be targeted by differentially expressed miRNAs between treatment groups and Pathway Studio Sub-Network Enrichment Analysis (SNEA) results. (XLSX)

S1 Fig Detailed Pathway Studio Sub-Network Enrichment Analysis (SNEA) results for over-represented GO-term processes related to glucose metabolism, as results visualized in Fig 7. (PDF)

## References

[1] Abbott JC, Dill LM. Patterns of Aggressive Attack in Juvenile Steelhead Trout (Salmo gairdneri). Can J Fish Aquat Sci. 1985 11 1;42(11):1702–6.

[2] Elliott JM. Mechanisms Responsible for Population Regulation in Young Migratory Trout, Salmo trutta. III. The Role of Territorial Behaviour. Journal of Animal Ecology. 1990;59(3):803–18.

[3] Metcalfe NB. Intraspecific variation in competitive ability and food intake in salmonids: consequences for energy budgets and growth rates J Fish Biol. 1986 5 28: 525–531.

[4] DiBattista JD, Levesque HM, Moon TW, Gilmour KM. Growth Depression in Socially SS Rainbow Trout Oncorhynchus mykiss: More than a Fasting Effect. Physiological and Biochemical Zoology. 2006 7 1;79(4):675–87. 10.1086/504612 16826494

[5] Gilmour KM, DiBattista JD, Thomas JB. Physiological Causes and Consequences of Social Status in Salmonid Fish. Integr Comp Biol. 2005 4 1;45(2):263–73. 10.1093/icb/45.2.263 21676770

[6] Gilmour KM, Kirkpatrick S, Massarsky A, Pearce B, Saliba S, Stephany C-É, et al The Influence of Social Status on Hepatic Glucose Metabolism in Rainbow Trout Oncorhynchus mykiss. Physiological and Biochemical Zoology. 2012 7 1;85(4):309–20. 10.1086/666497 22705482

[7] Kostyniuk DJ, Culbert BM, Mennigen JA, Gilmour KM. Social status affects lipid metabolism in rainbow trout, Oncorhynchus mykiss. Am J Physiol Regul Integr Comp Physiol. 2018 3 21; 10.1152/ajpregu.00402.2017 PMC6139616 29561648

[8] Mommsen TP, Moon TW. Metabolic response of teleost hepatocytes to glucagon-like peptide and glucagon. J Endocrinol. 1990 7;126(1):109–18. 2166124 10.1677/joe.0.1260109

[9] Vijayan MM, Reddy PK, Leatherland JF, Moon TW. The effects of cortisol on hepatocyte metabolism in rainbow trout: a study using the steroid analogue RU486. Gen Comp Endocrinol. 1994 10;96(1):75–84. 10.1006/gcen.1994.1160 7843570

[10] Culbert BM, Gilmour KM. Rapid recovery of the cortisol response following social subordination in rainbow trout. Physiology & Behavior. 2016 10 1;164:306–13. 27317163 10.1016/j.physbeh.2016.06.012

[11] Polakof S, Panserat S, Soengas JL, Moon TW. Glucose metabolism in fish: a review. J Comp Physiol B, Biochem Syst Environ Physiol. 2012 12;182(8):1015–45. 10.1007/s00360-012-0658-7 22476584

[12] Moon TW. Glucose intolerance in teleost fish: fact or fiction? Comp Biochem Physiol B, Biochem Mol Biol. 2001 6;129(2–3):243–9. 11399456 10.1016/s1096-4959(01)00316-5

[13] Rottiers V, Näär AM. MicroRNAs in metabolism and metabolic disorders. Nat Rev Mol Cell Biol. 2012 3 22;13(4):239–50. 10.1038/nrm3313 22436747 PMC4021399

[14] Mennigen JA. Micromanaging metabolism-a role for miRNAs in teleost energy metabolism. Comp Biochem Physiol B, Biochem Mol Biol. 2016 9;199:115–25. 10.1016/j.cbpb.2015.09.001 26384523

[15] Mennigen JA, Martyniuk CJ, Seiliez I, Panserat S, Skiba-Cassy S. Metabolic consequences of microRNA-122 inhibition in rainbow trout, Oncorhynchus mykiss. BMC Genomics. 2014 1 27;15:70 10.1186/1471-2164-15-70 24467738 PMC3914182

[16] Kim Y-K, Kim B, Kim VN. Re-evaluation of the roles of DROSHA, Exportin 5, and DICER in microRNA biogenesis. Proc Natl Acad Sci U S A. 2016 3 29;113(13):E1881–9. 10.1073/pnas.1602532113 26976605 PMC4822641

[17] Heckmann L-H, Sørensen PB, Krogh PH, Sørensen JG. NORMA-Gene: A simple and robust method for qPCR normalization based on target gene data. BMC Bioinformatics. 2011 6 21;12(1):250. 21693017 10.1186/1471-2105-12-250 PMC3223928

[18] Ma H, Weber GM, Hostuttler MA, Wei H, Wang L, Yao J. MicroRNA expression profiles from eggs of different qualities associated with post-ovulatory ageing in rainbow trout (Oncorhynchus mykiss). BMC Genomics. 2015;16:201 10.1186/s12864-015-1400-0 25885637 PMC4374207

[19] Berthelot C, Brunet F, Chalopin D, Juanchich A, Bernard M, Noël B, et al The rainbow trout genome provides novel insights into evolution after whole-genome duplication in vertebrates. Nat Commun. 2014 4 22;5:3657 10.1038/ncomms4657 24755649 PMC4071752

[20] Juanchich A, Bardou P, Rué O, Gabillard J-C, Gaspin C, Bobe J, et al Characterization of an extensive rainbow trout miRNA transcriptome by next generation sequencing. BMC Genomics. 2016 3 1;17:164 10.1186/s12864-016-2505-9 26931235 PMC4774146

[21] Mennigen JA, Zhang D. MicroTrout: A comprehensive, genome-wide miRNA target prediction framework for rainbow trout, Oncorhynchus mykiss. Comp Biochem Physiol Part D Genomics Proteomics. 2016 12;20:19–26. 10.1016/j.cbd.2016.07.002 27494513

[22] Enright AJ, John B, Gaul U, Tuschl T, Sander C, Marks DS. MicroRNA targets in Drosophila. Genome Biol. 2003;5(1):R1 10.1186/gb-2003-5-1-r1 14709173 PMC395733

[23] Rehmsmeier M, Steffen P, Hochsmann M, Giegerich R. Fast and effective prediction of microRNA/target duplexes. RNA. 2004 10;10(10):1507–17. 10.1261/rna.5248604 15383676 PMC1370637

[24] Bartel DP. MicroRNAs: target recognition and regulatory functions. Cell. 2009 1 23;136(2):215–33. 10.1016/j.cell.2009.01.002 19167326 PMC3794896

[25] Peterson SM, Thompson JA, Ufkin ML, Sathyanarayana P, Liaw L, Congdon CB. Common features of microRNA target prediction tools. Front Genet. 2014;5:23 10.3389/fgene.2014.00023 24600468 PMC3927079

[26] Fan X, Kurgan L. Comprehensive overview and assessment of computational prediction of microRNA targets in animals. Brief Bioinformatics. 2015 9;16(5):780–94. 10.1093/bib/bbu044 25471818

[27] Witkos TM, Koscianska E, Krzyzosiak WJ. Practical Aspects of microRNA Target Prediction. Curr Mol Med. 2011 3;11(2):93–109. 10.2174/156652411794859250 21342132 PMC3182075

[28] Paneru BD, Al-Tobasei R, Kenney B, Leeds TD, Salem M. RNA-Seq reveals MicroRNA expression signature and genetic polymorphism associated with growth and muscle quality traits in rainbow trout. Scientific Reports. 2017 8 22;7(1):9078 10.1038/s41598-017-09515-4 28831113 PMC5567286

[29] Koganti PP, Wang J, Cleveland B, Ma H, Weber GM, Yao J. Estradiol regulates expression of miRNAs associated with myogenesis in rainbow trout. Molecular and Cellular Endocrinology. 2017 3 5;443:1–14. 10.1016/j.mce.2016.12.014 28011237

[30] Hofacker IL, Fontana W, Stadler PF, Bonhoeffer LS, Tacker M, Schuster P. Fast folding and comparison of RNA secondary structures. Monatsh Chem. 1994 2 1;125(2):167–88.

[31] Herrera BM, Lockstone HE, Taylor JM, Ria M, Barrett A, Collins S, et al Global microRNA expression profiles in insulin target tissues in a spontaneous rat model of type 2 diabetes. Diabetologia. 2010 6;53(6):1099–109. 10.1007/s00125-010-1667-2 20198361 PMC2860560

[32] Best C, Ikert H, Kostyniuk DJ, Craig PM, Navarro-Martin L, Marandel L, et al Epigenetics in teleost fish: From molecular mechanisms to physiological phenotypes. Comp Biochem Physiol B, Biochem Mol Biol. 2018 1 31 10.1016/j.cbpb.2018.01.006 29369794

[33] Wai T, Langer T. Mitochondrial Dynamics and Metabolic Regulation. Trends in Endocrinology & Metabolism. 2016 2;27(2):105–17. 26754340 10.1016/j.tem.2015.12.001

[34] Schrepfer E, Scorrano L. Mitofusins, from Mitochondria to Metabolism. Molecular Cell. 2016 3;61(5):683–94. 10.1016/j.molcel.2016.02.022 26942673

[35] Hernández-Alvarez MI, Paz JC, Sebastián D, Muñoz JP, Liesa M, Segalés J, et al Glucocorticoid Modulation of Mitochondrial Function in Hepatoma Cells Requires the Mitochondrial Fission Protein Drp1. Antioxidants & Redox Signaling. 2013 Aug;19(4):366–78. 22703557 10.1089/ars.2011.4269 PMC3700019

[36] Sebastian D, Hernandez-Alvarez MI, Segales J, Sorianello E, Munoz JP, Sala D, et al Mitofusin 2 (Mfn2) links mitochondrial and endoplasmic reticulum function with insulin signaling and is essential for normal glucose homeostasis. Proceedings of the National Academy of Sciences. 2012 4 3;109(14):5523–8. 10.1073/pnas.1108220109 PMC3325712 22427360

[37] Hanson RW, Reshef L. Regulation of phosphoenolpyruvate carboxykinase (GTP) gene expression. Annu Rev Biochem. 1997;66:581–611. 10.1146/annurev.biochem.66.1.581 9242918

[38] Vijayan MM, Raptis S, Sathiyaa R. Cortisol treatment affects glucocorticoid receptor and glucocorticoid-responsive genes in the liver of rainbow trout. Gen Comp Endocrinol. 2003 6 15;132(2):256–63. 12812773 10.1016/s0016-6480(03)00092-3

[39] Mommsen TP, Vijayan MM, Moon TW. Cortisol in teleosts: dynamics, mechanisms of action, and metabolic regulation. Reviews in Fish Biology and Fisheries. 1999 9 1;9(3):211–68.

[40] Marandel L, Seiliez I, Véron V, Skiba-Cassy S, Panserat S. New insights into the nutritional regulation of gluconeogenesis in carnivorous rainbow trout (Oncorhynchus mykiss): a gene duplication trail. Physiol Genomics. 2015 7;47(7):253–63. 10.1152/physiolgenomics.00026.2015 25901068

[41] Kittilson JD, Reindl KM, Sheridan MA. Rainbow trout (Oncorhynchus mykiss) possess two hormone-sensitive lipase-encoding mRNAs that are differentially expressed and independently regulated by nutritional state. Comp Biochem Physiol, Part A Mol Integr Physiol. 2011 1;158(1):52–60. 10.1016/j.cbpa.2010.09.010 20858550

[42] Bergan-Roller HE, Ickstadt AT, Kittilson JD, Sheridan MA. Insulin and insulin-like growth factor-1 modulate the lipolytic action of growth hormone by altering signal pathway linkages. Gen Comp Endocrinol. 2017 01;248:40–8. 10.1016/j.ygcen.2017.04.005 28410970

[43] Ma M, Yin Z, Zhong H, Liang T, Guo L. Analysis of the expression, function, and evolution of miR-27 isoforms and their responses in metabolic processes. Genomics. In Press 10.1016/j.ygeno.2018.08.004. 30145283

[44] Calo N, Ramadori P, Sobolewski C, Romero Y, Maeder C, Fournier M, et al Stress-activated miR-21/miR-21* in hepatocytes promotes lipid and glucose metabolic disorders associated with high-fat diet consumption. Gut. 2016;65(11):1871–81. 10.1136/gutjnl-2015-310822 27222533

[45] Xu J., Zhang R., Shen Y., Liu G., Lu X., Wu C.I., 2013a. The evolution of evolvability in microRNA target sites in vertebrates. Genome Res. 23, 1810–1816 10.1101/gr.148916.112 24077390 PMC3814881

[46] Cora’ D, Re A, Caselle M, Bussolino F. MicroRNA-mediated regulatory circuits: outlook and perspectives. Physical Biology. 2017 6 6;14(4):045001 10.1088/1478-3975/aa6f21 28586314

[47] Zhang H-M, Kuang S, Xiong X, Gao T, Liu C, Guo A-Y. Transcription factor and microRNA co-regulatory loops: important regulatory motifs in biological processes and diseases. Briefings in Bioinformatics. 2015 1 1;16(1):45–58. 10.1093/bib/bbt085 24307685

