# Correction: Social status regulates the hepatic miRNAome in rainbow trout: Implications for posttranscriptional regulation of metabolic pathways

**DOI:** 10.1371/journal.pone.0233827

**Published:** 2020-05-21

**Authors:** Daniel J. Kostyniuk, Dapeng Zhang, Christopher J. Martyniuk, Kathleen M. Gilmour, Jan A. Mennigen

The terms socially SD and socially SS appear incorrectly throughout the abstract. The correct terms are SD and SS.

There is an error in reference 4. The correct reference is: DiBattista JD, Levesque HM, Moon TW, Gilmour KM. Growth Depression in Socially Subordinate Rainbow Trout Oncorhynchus mykiss: More than a Fasting Effect Physiological and Biochemical Zoology. 2006 Jul 1;79(4):675–87. https://doi.org/10.1086/504612 PMID: 16826494.

Fig 7A incorrectly uses a different sample set. The authors have provided a corrected version of Fig 7 here. As a result of this error in Fig 7A, there is a reference to incorrect samples in the second sentence of the first paragraph of the SS status induced *pck1* transcript in rainbow trout liver subsection of the Results. The correct sentence is: The mRNA abundance of cytoplasmic *pck1* (F_2,15_ = 14.75, p = 0.0003), but not mitochondrial *pck2* (F_2,16_ = 2.84, p = 0.0880), was significantly higher in SS trout compared to both SI and SD rainbow trout (p < 0.0001).

The x-axis labels in Fig 5A–5E have been incorrectly swapped. The dark bar should be labeled ‘SD’ and the light bar should be labeled ‘SS.’ The authors have provided a corrected version of Fig 5 here.

**Fig 5:**
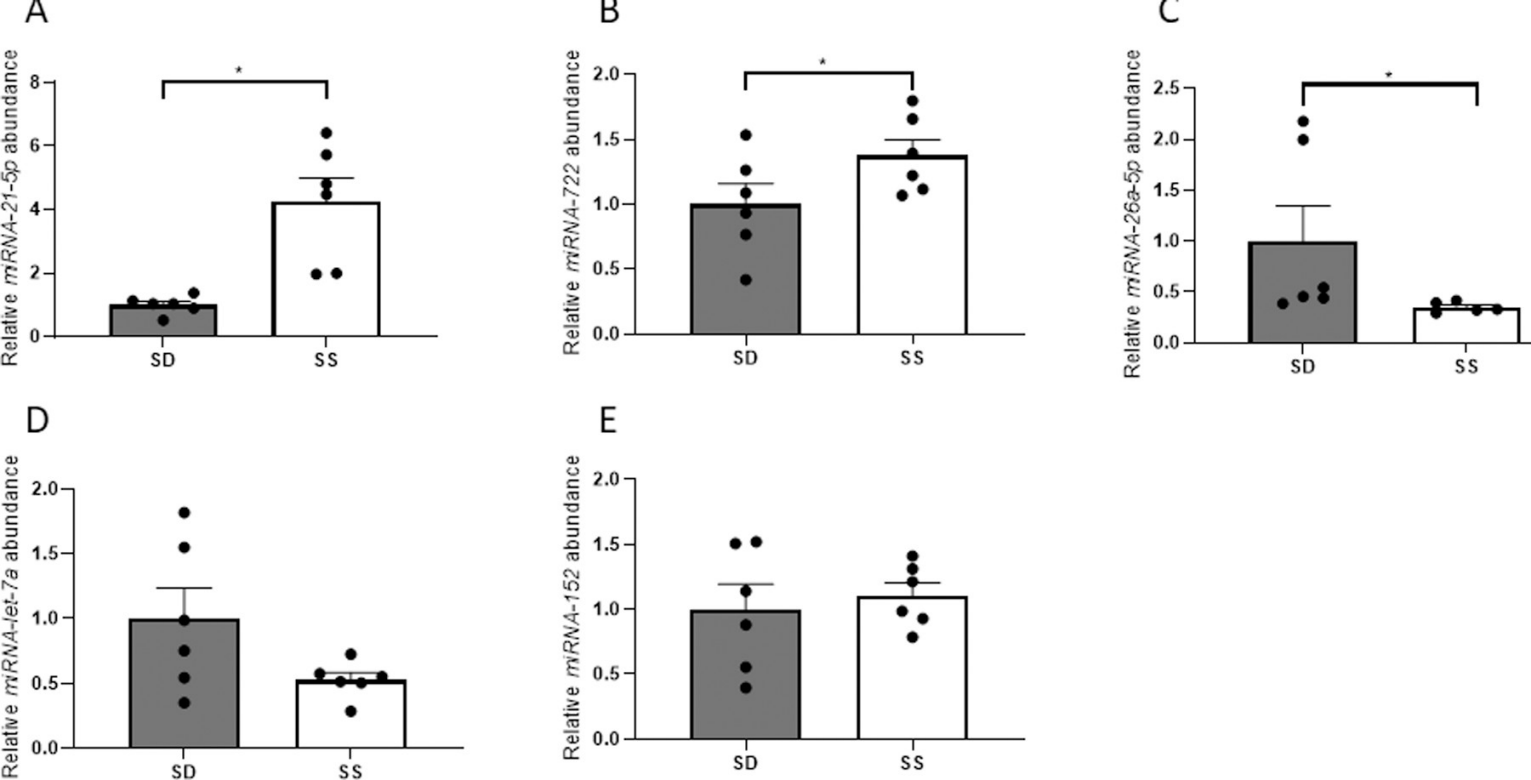
Relative steady-state abundance (+S.E.M.) of the most abundant differentially regulated hepatic miRNA, *miRNA-21-5p*, as well as *miRNA-722*, *miRNA-26a-5p*, *miRNA-let-7a* and *miRNA-152* in liver of SD and SS rainbow trout, *Oncorhynchus mykiss*. A one-tailed Welch’s t-test was used for analysis, and a p-value of p<0.05 was used as cut-off for significant effects.

**Fig 7:**
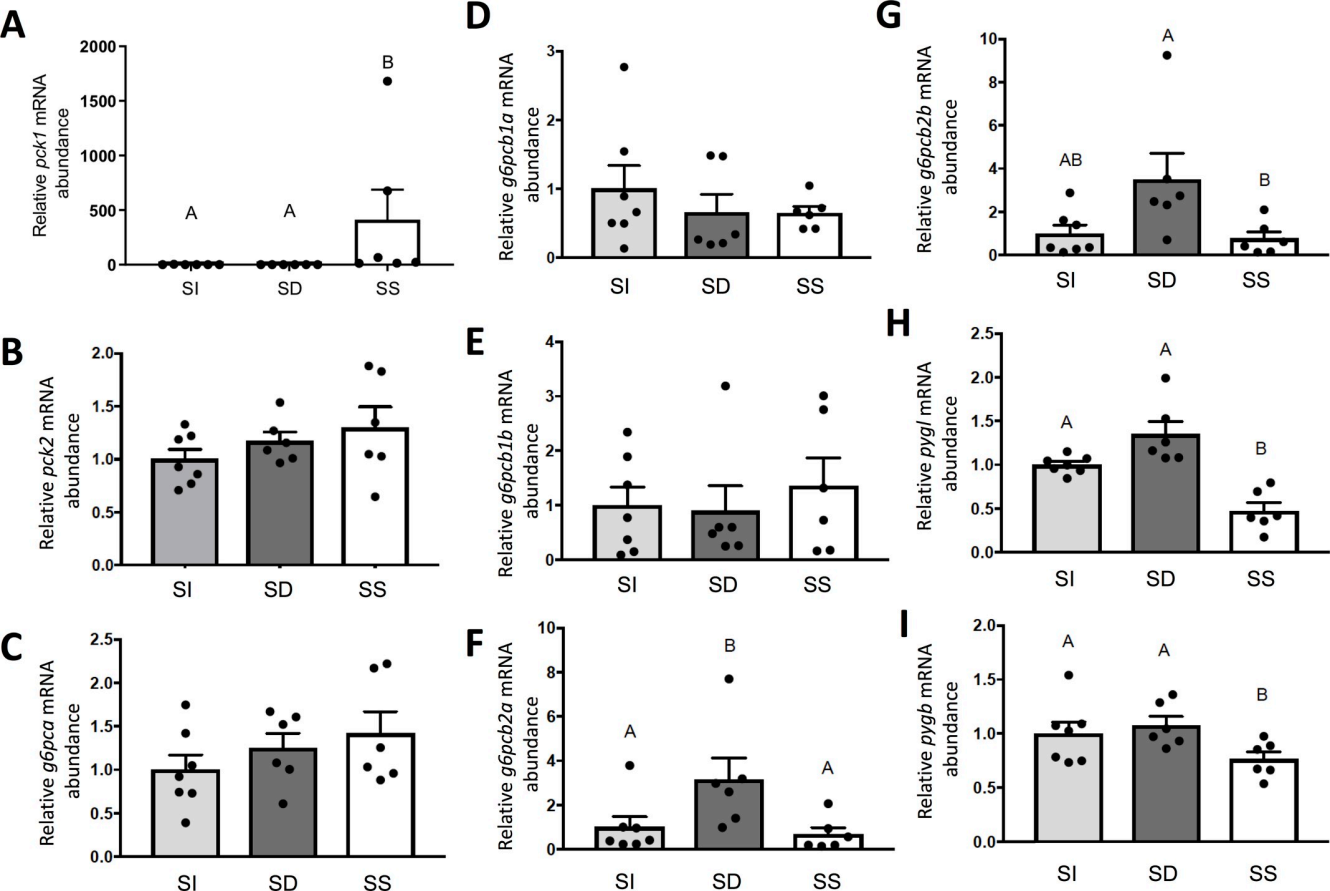
Steady state mRNA abundance (+S.E.M.) of genes involved in hepatic glucose metabolism, including gluconeogenic enzyme isoforms cytoplasmic phosphoenolpyruvate carboxykinase *pck1* (**A**), mitochondrial phosphoenolpyruvate carboxykinase *pck2* (**B**), gluconeogenic enzyme paralogues of glucose-6-phosphatase (**C-G**), and liver (**H**) and brain (**I**) isoforms of the glycogenolytic enzyme, glycogen phosphorylase. Data for SI, SD and SS rainbow trout (*Oncorhynchus muykiss*) were normalized using the Normagene algorithm, and then expressed relative to values for SI fish. A one-way ANOVAs followed by Tukey’s post-hoc was used for analysis. A p-value of p<0.05 was used as cut-off for significant effects.
