# Supplementary figures and images for: Social status regulates the hepatic miRNAome in rainbow trout: Implications for posttranscriptional regulation of metabolic pathways
Source: PLoS One. 2019 Jun 13;14(6):e0217978. doi: 10.1371/journal.pone.0217978 (PMC6563994; doi:10.1371/journal.pone.0217978)

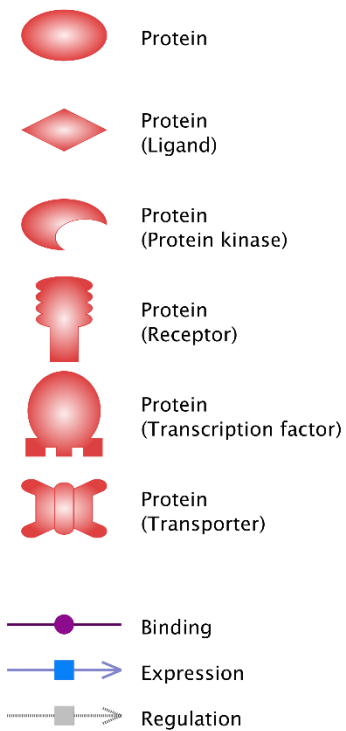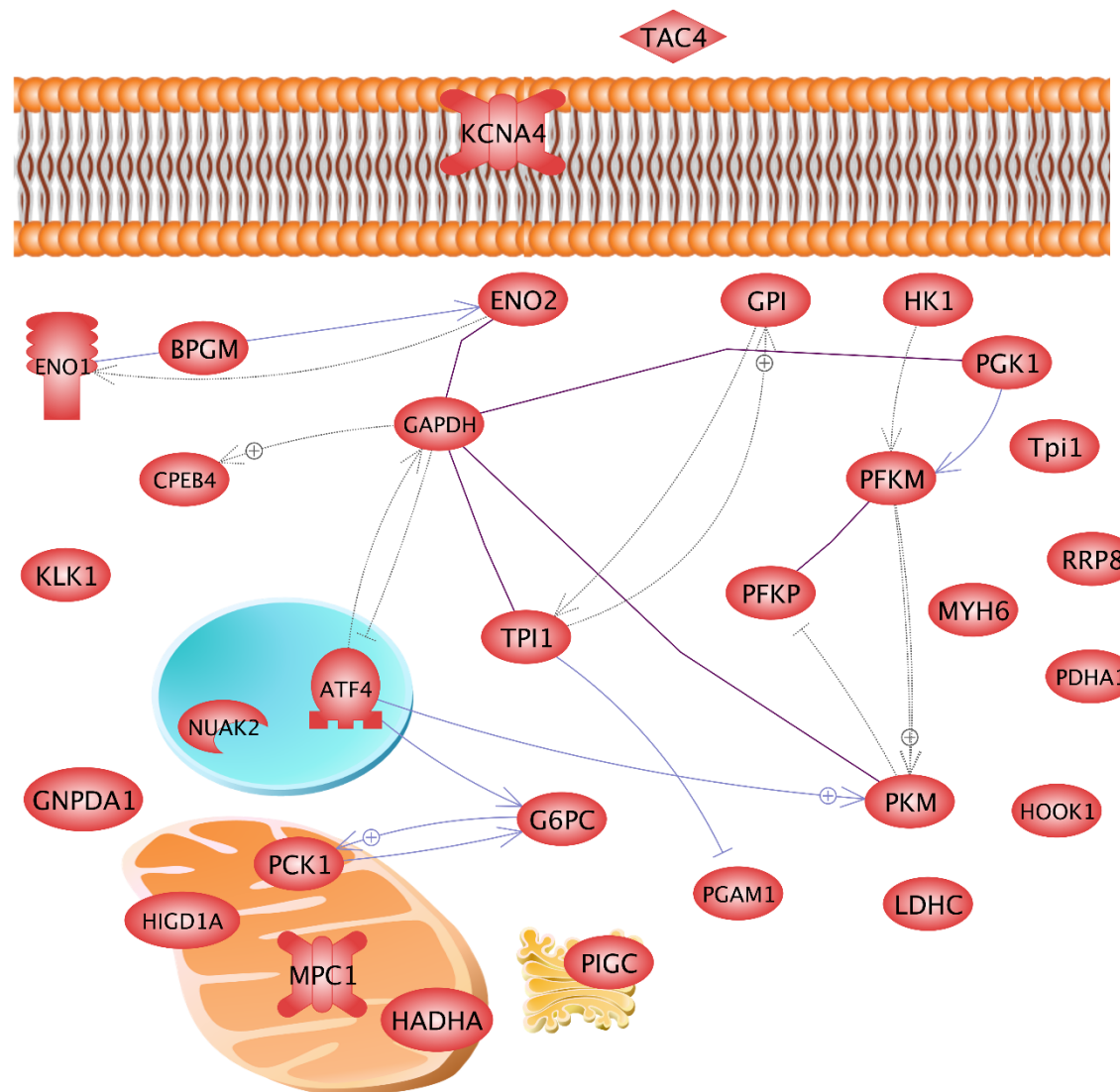

Glycolytic process  
GO:0006096

cellular response to glucose  
starvation GO:0042149

Gluconeogenesis  
GO:0006094

Supplement: S1 Fig — (PDF) [file pone.0217978.s006.pdf]
